# Gut *Parabacteroides distasonis*‐derived Indole‐3‐Acetic Acid Promotes Phospholipid Remodeling and Enhances Ferroptosis Sensitivity via the AhR‐FASN Axis in Bladder Cancer

**DOI:** 10.1002/advs.202504688

**Published:** 2025-06-25

**Authors:** Weijia Li, Wentai Shangguan, Wenxue Huang, Jie Zhao, Yuexuan Zhu, Ming Xie, Yao Yu, Qishen Yang, Jun Zheng, Lin Yang, Qi Sun, Leqian Li, Xinhang Shi, Yongyuan Xiao, Hai Huang, Bisheng Cheng, Peng Wu

**Affiliations:** ^1^ Department of Urology Nanfang Hospital Southern Medical University Guangzhou Guangdong 510515 P. R. China; ^2^ NMPA Key Laboratory for Research and Evaluation of Drug Metabolism Guangdong Provincial Key Laboratory of New Drug Screening School of Pharmaceutical Sciences Southern Medical University Guangzhou 510515 China; ^3^ Department of Urology Sun Yat‐sen Memorial Hospital Sun Yat‐sen University Guangzhou 510120 China

**Keywords:** aryl hydrocarbon receptor, bladder cancer, ferroptosis, indole‐3‐acetic, *Parabacteroides distasonis*

## Abstract

The development of bladder cancer is a complex, multistep process influenced by both genetic and environmental factors, but its exact etiology remains unclear. Increasing evidence suggests that gut microbiota dysregulation can impact the initiation and progression of cancers, including colorectal cancer, breast cancer, and pancreatic cancer. This study observes that the beneficial gut bacterium *Parabacteroides distasonis* (*P. distasonis*) is significantly decreased in bladder cancer patients, and its low abundance is strongly associated with poor prognosis. *P. distasonis* is a next‐generation probiotic which plays a vital role in human health. Furthermore, this work finds that *P. distasonis* culture medium inhibits metastasis and proliferation of bladder cancer cells in vitro and in vivo. This work identifies that indole‐3‐acetic acid (3‐IAA) produced by *P. distasonis* exerting similar tumor‐suppressive effects. Mechanistically, 3‐IAA activates the aryl hydrocarbon receptor (AhR), which in turn downregulates fatty acid synthase (FASN) transcription in bladder cancer cells. Notably, the reduction of FASN expression decreases the ratio of MUFAs to PUFAs, thereby increasing ferroptosis sensitivity in bladder cancer cells. Collectively, this study demonstrates that *P. distasonis*‐derived 3‐IAA can inhibit bladder cancer metastasis and proliferation, highlighting the AhR‐FASN axis as a promising therapeutic target to inhibit bladder cancer progression.

## Graphical abstract

Indole‐3‐acetic acid (3‐IAA) derived from gut *P. distasonis* binds to AhR in bladder cancer cells and downregulates FASN transcription, thereby increasing sensitivity to ferroptosis and suppressing the development of bladder tumors. Specifically, 3‐IAA treatment leads to an increase in the proportion of PC/PE‐PUFAs and a decrease in the proportions of PC/PE‐MUFAs, creating an imbalance that induces ferroptosis in cancer cells.

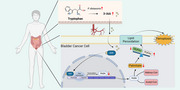

## 1 Introduction

Bladder cancer is the ninth most commonly diagnosed cancer globally, with an estimated 614 000 new cases and 220 000 deaths reported in 2022^[^ ^1^ ^]^ Bladder cancer can be classified into non‐muscle‐invasive bladder cancer (NMIBC) and muscle‐invasive bladder cancer (MIBC) based on the depth of invasion. MIBC accounts for ≈1% of newly diagnosed bladder cancer cases, with ≈15%–50% of NMIBC cases eventually progress to MIBC. The 20‐year overall survival rate for patients with MIBC ranges from 5% to 40%.^[^ ^2^ ^]^ The development of bladder cancer is a complex, multistep process influenced by both genetic and environmental factors, but its exact etiology remains unclear.^[^ ^3^, ^4^ ^]^

The human body harbors trillions of microbial cells, and their synergistic interactions are crucial for vital physiological processes.^[^ ^5^ ^]^ When microbial populations in the gut reach their highest density, they form a complex community known as the gut microbiota.^[^ ^6^ ^]^ The gut microbiota plays crucial roles in host growth and development, metabolism, and immune regulation. Dysbiosis of the human gut microbiota is associated with various diseases, and increasing evidence suggests that microbial dysregulation can impact the initiation and progression of cancers, including colorectal cancer, breast cancer, and pancreatic cancer.^[^ ^7^ ^]^ A recent study revealed that the metabolism of carcinogens by the gut microbiota may contribute to chemically induced carcinogenesis in the bladder.^[^ ^8^ ^]^

The gut microbiota influences disease primarily through its derived metabolites, including indole metabolites. Indole metabolites are generated through the indole pathway of tryptophan metabolism, a process predominantly regulated by enzymes encoded and expressed by the gut microbiota.^[^ ^9^ ^]^ Recent studies have revealed that indole metabolites can directly affect cancer. For example, ILA produced by *Lactobacillus gallinarum* inhibits colorectal cancer cell proliferation and promotes apoptosis in vitro.^[^ ^10^ ^]^ IDA derived from *Peptostreptococcus anaerobius* acts as an AhR ligand and increases ALDH1A3/NADH levels, inhibiting tumor ferroptosis.^[^ ^11^ ^]^ This finding suggests that microbial metabolites can modulate ferroptosis in cancer cells. *Prevotella copri* extensively consumes host tryptophan, leading to a reduction in IPYA accumulation in the tumor microenvironment, which suppresses the growth of breast and cervical cancer cells.^[^ ^12^ ^]^ However, whether gut microbiota‐derived metabolites can similarly influence bladder cancer progression via pathways such as ferroptosis remains unknown.

Ferroptosis is a nonapoptotic form of cell death, that is distinct from apoptosis and autophagy, and is characterized by the accumulation of lipid peroxides.^[^ ^13^ ^]^ Free intracellular iron or iron‐containing enzymes react with oxygen and lipids containing polyunsaturated fatty acids (PUFAs), generating high levels of membrane lipid peroxides, which leads to ferroptosis.^[^ ^14^ ^]^ Tumor cells can inhibit ferroptosis by suppressing peroxide levels, with the key being the inhibition of PUFAs, particularly the levels of PUFA‐phospholipids. The accumulation of monounsaturated fatty acids (MUFAs) and saturated fatty acids (SFAs) protects cancer cells from ferroptosis.^[^ ^15^, ^16^ ^]^ Therefore, the expression of enzymes involved in the metabolism of PUFAs may regulate the sensitivity of cancer cells to ferroptosis.^[^ ^17^ ^]^ Fatty acid synthase (FASN) is a pivotal enzyme in lipid metabolism, primarily catalyzing the synthesis of saturated fatty acids from acetyl‐CoA and malonyl‐CoA, which is central to fatty acid biosynthesis, with its products serving as precursors for phospholipid synthesis, thereby regulating the composition and function of cell membranes. FASN elevates cellular levels of SFAs and MUFAs and also enables the conversion of essential fatty acids into more highly unsaturated fatty acids, thereby reducing the cell's sensitivity to ferroptosis.^[^ ^18^ ^]^

To address the above knowledge gap, in this study we aimed to explore the impact of gut microbiota‐derived metabolites on bladder cancer and the underlying mechanisms. We demonstrated that indole‐3‐acetic acid (3‐IAA) derived from gut *Parabacteroides distasonis* (*P. distasonis)* binds to aryl hydrocarbon receptor (AhR) in bladder cancer cells and downregulates FASN transcription, thereby increasing sensitivity to ferroptosis and suppressing the development of bladder tumors. Specifically, 3‐IAA treatment leads to an increase in the proportion of PC/PE‐PUFAs and a decrease in the proportions of PC/PE‐MUFAs, creating an imbalance that induces ferroptosis in cancer cell. Our findings reveal a novel gut microbiota‐host interaction mechanism and suggest that the AhR‐FASN axis could serve as a therapeutic target in bladder cancer.

## 2 Results

### 2.1 The abundance of *P. distasonis* is Associated with the Prognosis of Bladder Cancer Patients

**Figure 1:**
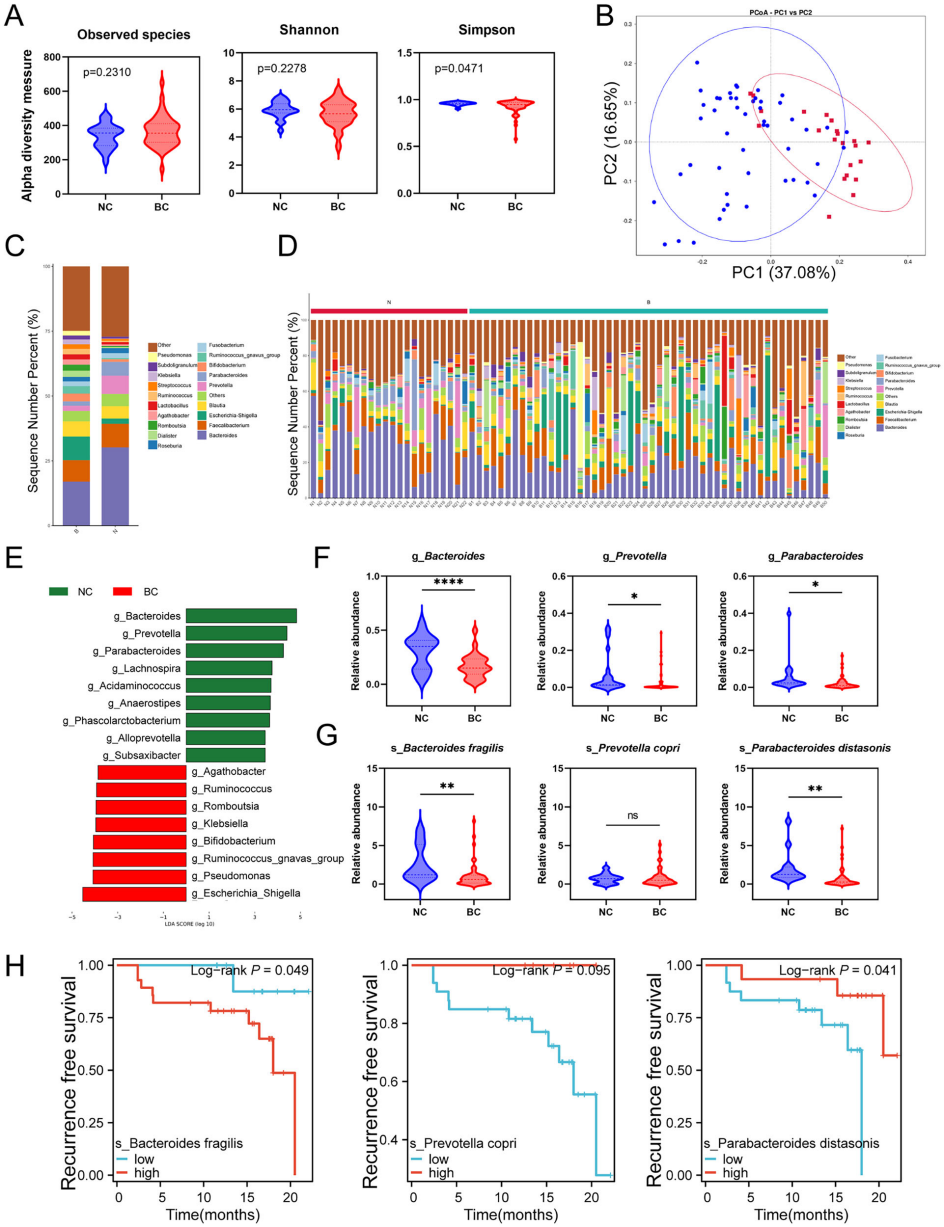
The abundance of *P. distasonis* is associated with the prognosis of BCa patients. Alpha diversity between the BC and NC groups (Observed species, Shannon, and Simpson indices). B) PCoA using the weighted UniFrac distance of beta diversity. C,D) Taxonomic composition of the gut microbiota at the genus level. E) LDA score of features with different abundances between BC and NC groups. The criteria for differential feature is an LDA score >3. F) Relative abundances of *Bacteroides, Prevotella, and Parabacteroides* at the genus level. G) Relative abundances of *Bacteroides fragilis, Prevotella copri*, and *P. distasonis*. H–J)Kaplan‒Meier curves for reccurence free survival of BCa patients with high versus low expression of *Bacteroides fragilis, Prevotella copri*, and *P. distasonis*. Data are presented as mean ± SEM. *p < 0.05, **p < 0.01, ***p < 0.001; ns: not significant. *P. distasonis*: *Parabacteroides distasonis*; PCoA: principal coordinate analysis; LDA: linear discriminant analysis, BC: bladder cancer group.

To investigate the differences in the gut microbiota between bladder cancer patients and healthy controls, we collected fecal samples from 50 bladder cancer patients (BC group) and 22 matched controls (NC group) at our center between July 2018 and October 2021, based on exclusion criteria, for 16s rDNA sequencing.^[^ ^19^ ^]^ We first assessed tumor‐associated microbial diversity using various methods, including the observed species, the Shannon, and the Simpson indices. The results showed that that the alpha diversity of the gut microbiome, defined as the abundance and diversity of species within each fecal sample, was significantly higher in NC patients compared to BC patients (**Figure** **1**A). We then used OTU‐based weighted UniFrac distance PCoA to identify significant differences in the gut microbiota composition between the two groups (Figure 1B). At the genus level, the microbial communities in both the NC and BC groups consisted primarily of *Bacteroides, Faecalibacterium, Parabacteroides*, and *Escherichia‐Shigella* (Figure 1C,D).

Next, we investigated the relationship between the gut microbiota and bladder cancer patients. LEfSe analysis revealed that, at the genus level, fecal *Bacteroides*, *Prevotella* and *Parabacteroides* were significantly enriched and ranked among the top three genera in the NC group, whereas fecal *Escherichia‐Shigella*, *Pseudomonas*, and *Ruminococcus* were among the top three genera in the BC group (Figure 1E). Additionally, the relative abundances of fecal *Bacteroides*, *Prevotella*, and *Parabacteroides* were significantly different (Figure 1F). Accordingly, fecal samples were collected from 40 bladder cancer patients and 14 matched healthy controls based on the inclusion and exclusion criteria. The patients' detailed baseline characteristics are listed in Table S1, Supporting Information. Using quantitative PCR (qPCR), we measured the relative abundances of three representative species: *Bacteroides fragilis*, *Prevotella copri*, and *Parabacteroides distasonis*. We detected significant differences in the relative abundances of *Bacteroides fragilis* and *P. distasonis*, both of which were enriched in the NC group, whereas no differences were observed in the relative abundance of *Prevotella copri* (Figure 1G). We then classified BC patients into high‐expression and low‐expression groups for *Bacteroides fragilis*, *P. distasonis*, and *Prevotella copri* based on optimal cutoff values. Kaplan‐Meier survival analysis revealed that patients with higher fecal *Bacteroides fragilis* expression exhibited significantly shorter recurrence‐free survival (RFS), whereas those with elevated *P. distasonis* expression had notably longer RFS. No significant differences in RFS were observed within the *Prevotella copri* group (Figure 1H). These findings suggest that increased abundance of *P. distasonis* is associated with improved prognosis in bladder cancer patients.

### 2.2 *P. distasonis* Culture Medium Inhibits the Growth and Migration of Bladder Cancer Cell

**Figure 2:**
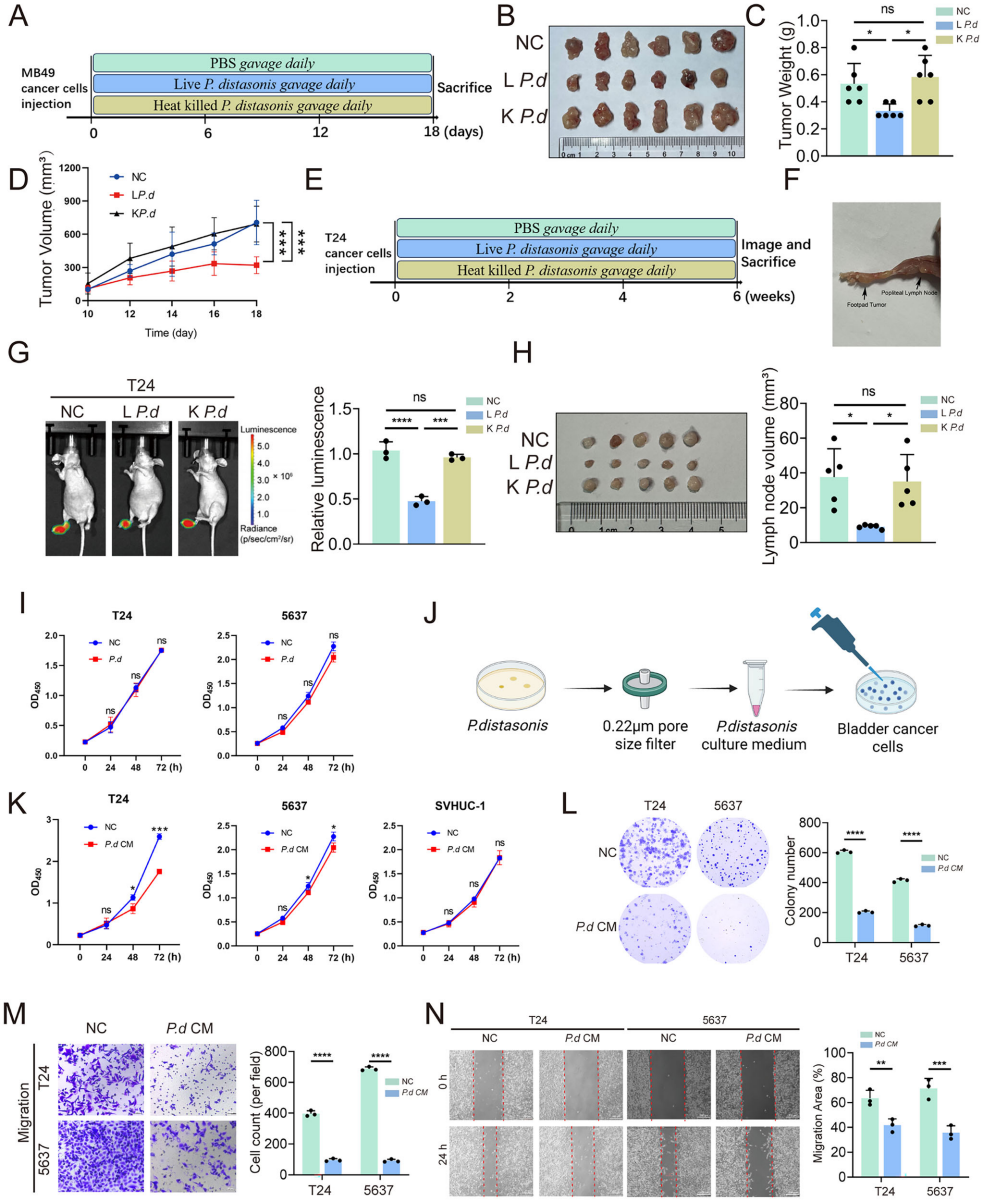
*P. distasonis* culture medium inhibits the growth and migration of bladder cancer cells. A) Schematic diagram of the subcutaneous tumor model established in C57BL/6 mice. B) Images of bladder tumors, C) statistical charts of the tumor weights and D) tumor volume growth curves in mice. (n = 6) E) Schematic representation of the popliteal LN metastasis model established in nude mice. F) The popliteal LN metastasis model and G) representative bioluminescence images (n = 3). H) Histogram analysis of popliteal metastatic LNs and their volumes in the popliteal LN metastasis model established by different experimental groups. (n = 5) I) The proliferation of T24 and 5637 cells co‐cultured with *P. distasonis* was evaluated using CCK‐8 assays. J) Schematic illustration of bladder cancer cells treatment with *P. distasonis* culture medium. K) The proliferation of T24, 5637 and SCHUC‐1 cells co‐cultured with *P. d* CM, was evaluated using CCK‐8 assays. L) Colony formation of T24 and 5637 cells co‐cultured with *P. d* CM or NC M) Representative images of migration assays and a histogram showing the number of migrating T24 and 5637 cells co‐cultured with *P.d* CM or NC. N) Representative images of wound healing assays and a histogram showing the cell migration distance in T24 and 5637 cells co‐cultured with *P.d* CM or NC. Data are presented as mean ± SEM. *p < 0.05, **p < 0.01, ***p < 0.001, ns: not significant. *P. distasonis*: *Parabacteroides distasonis*; LN: lymph node; L *P. d*: Live *P. distasonis;* K *P. d*: Heat killed *P. distasonis; P.d* CM: *P. distasonis* culture medium; NC: broth control.

To validate the effect of *P. distasonis* on bladder cancer, we used MB49 cells to establish a subcutaneous tumor model in C57BL/6 mice. Based on the experimental schematic, these mice were orally gavaged with live *P. distasonis* (L *P. d*), heat‐killed *P. distasonis* (K *P. d)* or PBS for 18 days (**Figure** **2**A). Compared with the NC and K *P. d* groups, tumor volume and size were significantly reduced in the L *P. d* group. (Figure 2B–D). Moreover, the expression of the proliferation marker Ki‐67 in the L *P. d* group was significantly lower than that in the NC and K *P. d* groups (Figure S1A, Supporting Information). Next, we established a popliteal lymph node (LN) metastasis model using T24 cells expressing firefly luciferase. Mice were then randomly divided into groups and orally gavaged with L *P. d*, K *P. d*, or PBS daily for 6 weeks (Figure 2E). The LN metastasis model was successfully established (Figure 2F). Consistent with the results in the subcutaneous tumor model mice, the L *P. d* group presented significantly reduced luminescence in the footpad and popliteal LNs (Figure 2G). Moreover, compared with other two groups, the volumes of the popliteal LNs were smaller in the L *P. d* group(Figure 2H). Systemic toxicity of *P. distasonis* in mice was evaluated by assessing serum biochemical parameters (AST, ALT, BUN, and CRE) (Figure S2A–F, Supporting Information) and performing hematoxylin and eosin (H&E) staining of major organs (Figure S2G, Supporting Information), including the heart, liver, spleen, lung, and kidney. The results indicated that no significant organ damage or inflammation was observed at the administered dosage. This preliminary evidence indicated that live *P. distasonis* can inhibit tumor growth and lymphatic metastasis in vivo in bladder cancer cells.

To further demonstrate the tumor‐suppressive effects of *P. distasonis* in vitro, T24 and 5637 human bladder cancer cells were first co‐cultured with *P. distasonis*, and their proliferative activity was subsequently assessed. However, compared with the broth control group, no significant differences were observed in the proliferative activity of T24 and 5637 cells co‐cultured with *P. distasonis* (Figure 2I). These findings suggest the tumor‐suppressive effect of *P. distasonis* may not be due to the bacteria itself, but rather to its metabolites. To test this hypothesis, human bladder cancer cells were treated with *P. distasonis* culture medium (*P. d* CM) or broth control (NC) (Figure 2J). Treatment with *P. d* CM significantly suppressed the proliferation of T24 and 5637 cells, while having no effect on the proliferation of the normal bladder epithelial cell line SVHUC‐1 (Figure 2K). Similar results were observed in the colony formation assay (Figure 2L). In addition, in the wound healing and migration assays, we found that *P. d* CM treatment significantly inhibited the migration speed and number of migrating bladder cancer cells (Figure 2M,N). In summary, metabolites derived from *P. distasonis* can inhibit the tumor growth and migration of bladder cancer tumors.

### 2.3 3‐IAA is a Crucial Metabolite Produced by *P. distasonis*

**Figure 3:**
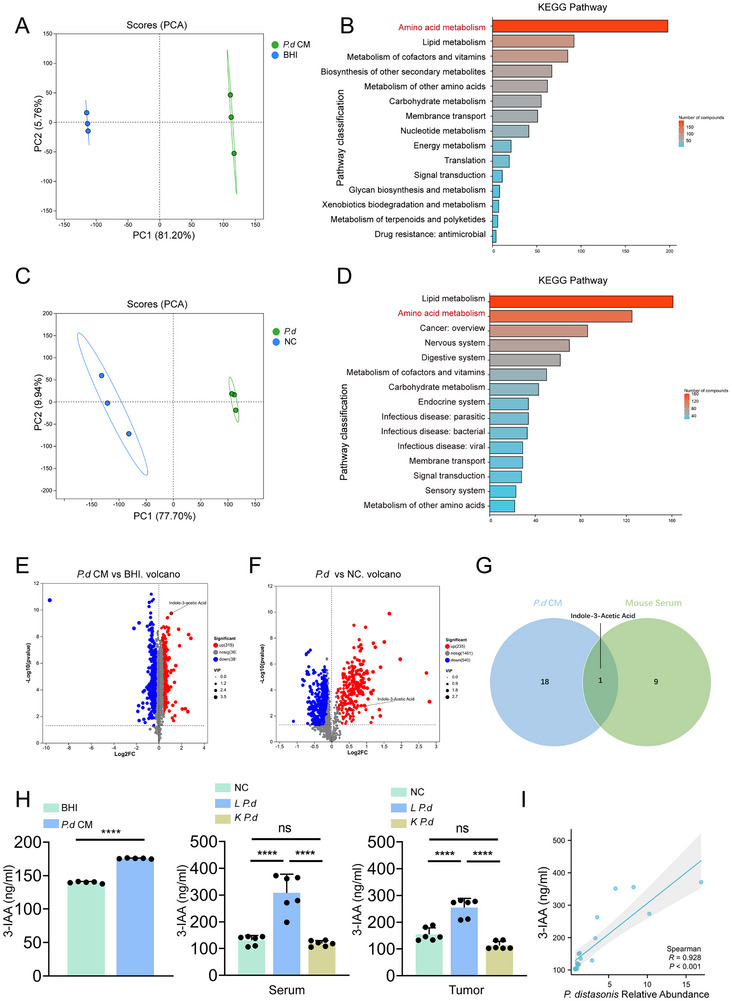
3‐IAA is a crucial metabolite produced by *P. distasonis*. A) The unweighted UniFrac distance of β‐diversity between the *P. d* CM and NC groups (n = 3). B) KEGG enrichment analysis revealed the pathways significantly enriched in the *P. d* CM group compared with the NC group. C) The unweighted UniFrac distance of β‐diversity between the *P. distasonis* and NC groups (n = 3). D) KEGG enrichment analysis revealed the pathways significantly enriched in the *P. distasonis* and NC groups. E) Volcano plots illustrating differentially abundant metabolites between the *P. d* CM and the NC group. F) Volcano plots illustrating the differentially abundant metabolites between the *P. distasonis* and NC groups. G) Venn diagram showing the intersection between the amino acid metabolites from the *P. d* CM and the serum from *P. distasonis* group. H) 3‐IAA levels in *P. d* CM, animal serum and tumors. I) Pearson correlation analysis of the levels of *P. distasonis* and 3‐IAA in mice. Data are represented as mean ± SEM. *p < 0.05, **p < 0.01, ***p < 0.001; ns: not significant. 3‐IAA: indole‐3‐acetic acid; *P. distasonis*: *Parabacteroides distasonis*; *P. d* CM: *P. distasonis* culture medium.

To identify key tumor‐suppressive metabolites produced by *Parabacteroides distasonis*, untargeted LC‒MS/MS analysis was performed on *P. d* CM and serum from mice gavaged with *P. distasonis*. Principal component analysis (PCA) revealed distinct metabolite clustering between *P. d* CM and the broth control (**Figure** **3**A). Kyoto Encyclopedia of Genes and Genomes analysis further revealed that the differentially abundant metabolites were enriched primarily in amino acid metabolism pathways (Figure 3B). Similarly, in the mouse serum, distinct metabolite clustering was observed from the *P. distasonis* and NC groups (Figure 3C). KEGG pathway enrichment analysis revealed that the amino acid metabolism pathway ranked second, which was consistent with the pathways enriched in *P. d* CM (Figure 3D). The volcano plot revealed that 235 metabolites were increased in *P. d* CM, and 315 metabolites were upregulated in the *P. distasonis* group mouse serum (Figure 3E,F). Therefore, we focused on the overlap of upregulated amino acid derivatives in *P. d* CM and in serum from mice gavaged with *P distasonis*, and visualized them using a Venn diagram. The Venn diagram revealed that 18 amino acid derivatives were exclusively present in *P. d* CM, whereas 9 amino acid derivatives were exclusively present in *P. distasonis* group mouse serum. Indole‐3‐actice acid (3‐IAA) was the only amino acid derivative shared by both groups (Figure 3G). ELISAs were used to measure the concentration of 3‐IAA in *P. d* CM, mouse serum and tumors. The concentration of 3‐IAA in *P. d* CM was significantly higher than that in the broth control. Similarly, elevated 3‐IAA levels were also observed in the serum and tumor tissues of the *P. distasonis* group. (Figure 3H). Moreover, the relative abundance of PD was positively correlated with the concentration of 3‐IAA (Figure 3I). These findings suggest that 3‐IAA may be a key metabolite derived from *P. distasonis*. We next sought to validate the inhibitory effects of 3‐IAA on bladder cancer in both in vivo and in vitro models.

### 2.4 3‐IAA Derived from *P. distasonis* can Exert Tumor‐Suppressive Effects

**Figure 4:**
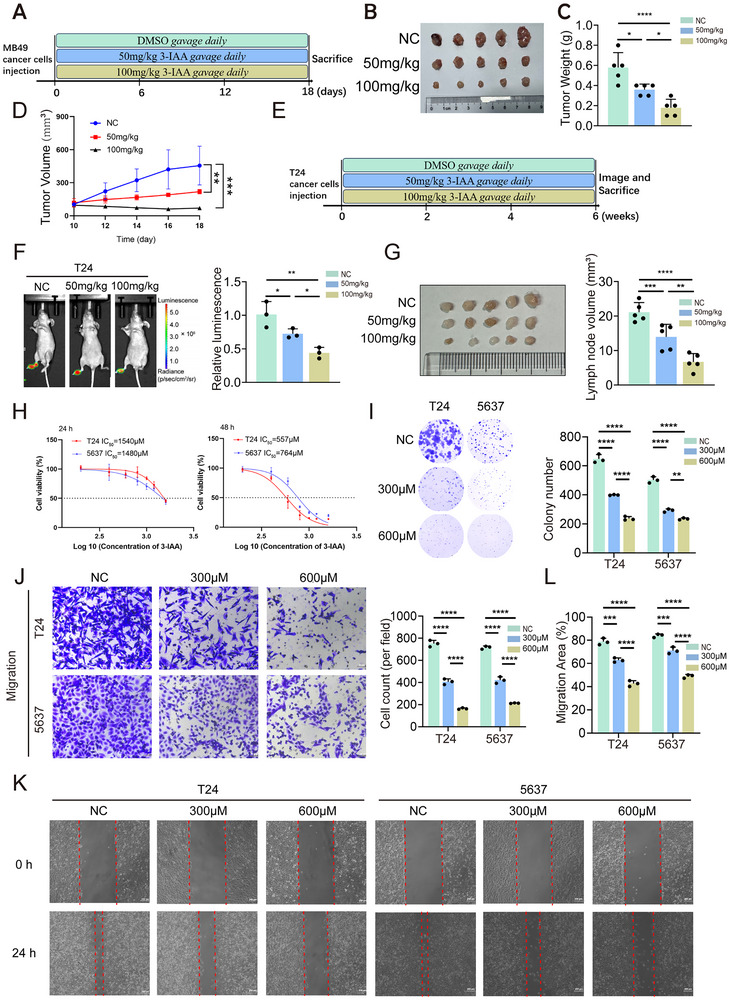
3‐IAA derived from *P. distasonis* can exert tumor‐suppressive effects. A) Schematic diagram of the subcutaneous tumor model established in C57BL/6 mice. B) Images of bladder tumors, C) statistical charts of the tumor weights and D) tumor volume growth curves in mice. (n = 5) E) Schematic representation of the establishment of a popliteal LN metastasis model in nude mice. F) Representative bioluminescence images. (n = 3) G) The popliteal LN metastasis model and histogram analysis of metastatic popliteal LNs and their volumes in models established by different experimental groups. (n = 5) H) The proliferation of T24 and 5637 cells treated with different concentrations of 3‐IAA for 24 or 48 h was evaluated using CCK‐8 assays. IC₅₀ values are shown. I) Colony formation of T24 and 5637 cells treated with different concentrations of 3‐IAA (300 µM and 600 µM) or DMSO. J) Representative images of migration assays and a histogram showing the number of migrating in T24 and 5637 cells treated with different concentrations of 3‐IAA (300 and 600 µM) or DMSO. K) Representative images of wound healing assays and L) a histogram showing the cell migration distance in T24 and 5637 cells treated with different concentrations of 3‐IAA (300 and 600 µM) or DMSO. Data are presented as mean ± SEM. *p < 0.05, **p < 0.01, ***p < 0.001; ns: not significant. 3‐IAA: indole‐3‐acetic acid; LN: lymph node; IC_50:_ half maximal inhibitory concentration.

We first investigated the tumor‐suppressive effects of 3‐IAA in vivo. We implanted MB49 cells subcutaneously into C57BL/6 mice. Based on the experimental schematic, mice were administered 3‐IAA or DMSO by oral gavage (**Figure** **4**A). Oral gavage of 3‐IAA significantly suppressed tumor growth in a dose‐dependent manner (Figure 4B–D). Expression of the proliferation marker Ki‐67 was significantly lower in the 3‐IAA‐treated groups compared to the NC group, and this effect was dose‐dependent (Figure S3A, Supporting Information). In the popliteal LN metastasis model established using T24 cells expressing firefly luciferase, oral administration of 3‐IAA significantly reduced bioluminescence signals in the footpads and popliteal LNs, with more pronounced reductions observed at higher concentrations (Figure 4E,F) Additionally, compared with the control group, the volume of popliteal LNs in the 3‐IAA‐treated group was significantly decreased in a dose‐dependent manner (Figure 4G).

We next assessed the inhibitory effects of 3‐IAA on bladder cancer cells in vitro. 3‐IAA exhibited potent anti‐proliferative activity against bladder cancer cell lines. After 24 h of 3‐IAA treatment, the half‐maximal inhibitory concentration (IC_50_) was 1540 µM for T24 cells and 1480 µM for 5637 cells. After 48 h of treatment, the IC_50_ values decreased to 557 µM for T24 cells and 764 µM for 5637 cells (Figure 4H). Based on these results, 3‐IAA at concentrations of 300and 600 µM for 48 h was selected for subsequent experiments. A colony formation assay demonstrated that 3‐IAA inhibits the clonogenic ability of bladder cancer cells in a dose‐dependent manner (Figure 4I). Furthermore, wound healing and transwell migration assays revealed that 3‐IAA significantly inhibited both the migration speed and the number of migrating bladder cancer cells in a dose‐dependent manner (Figure 4J–L). These findings suggest that 3‐IAA inhibits tumor growth in vivo in a dose‐dependent manner, as well as proliferation and migration in vitro. These findings indicate that 3‐IAA can exert tumor‐suppressive effects.

### 2.5 AhR as a Downstream Receptor to 3‐IAA is Associated with Favorable Prognosis in Bladder Cancer

**Figure 5:**
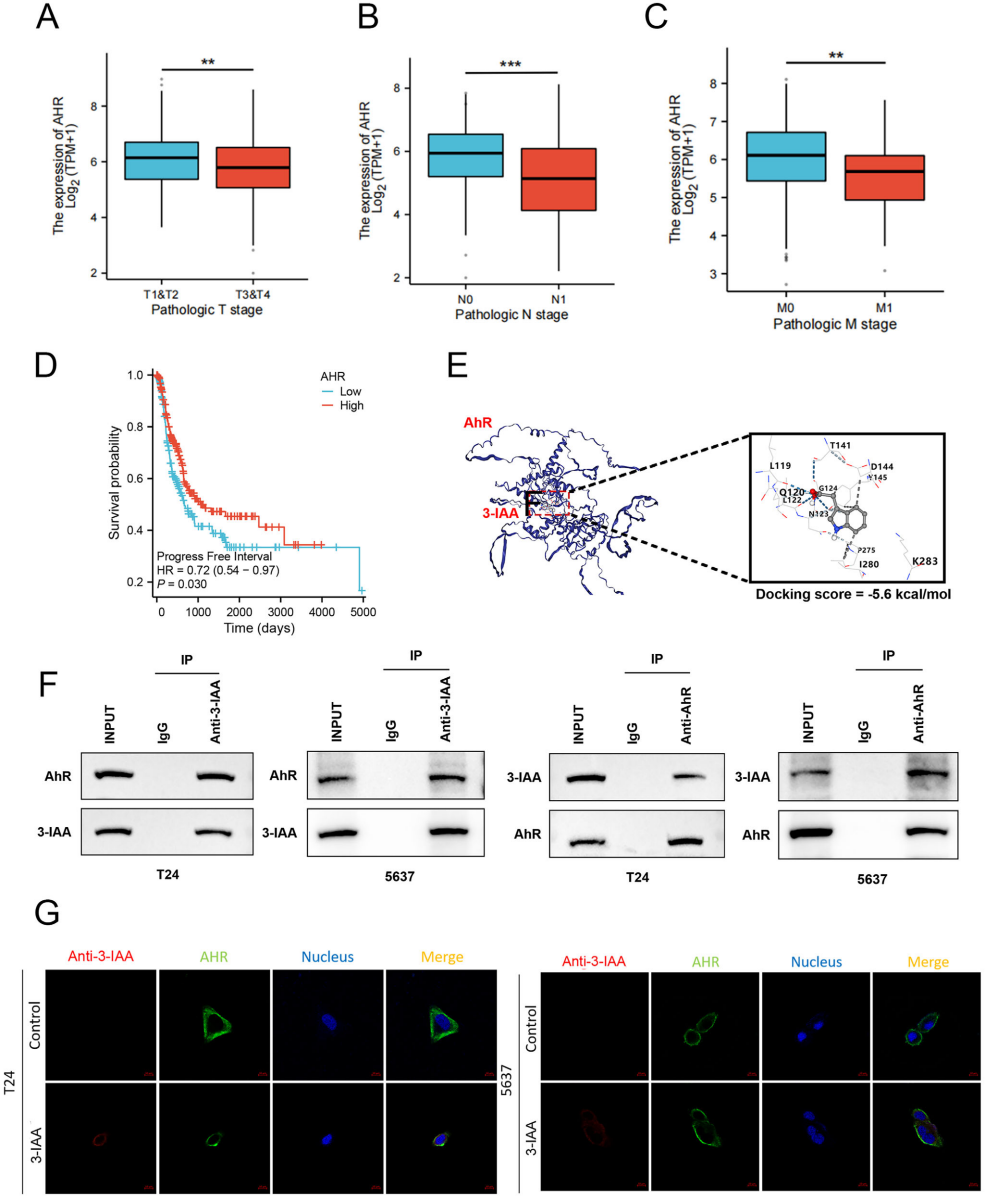
AhR as a downstream receptor to 3‐IAA is associated with favorable prognosis in bladder cancer. A) Analysis of AhR expression in the T1+T2 and T3+T4 groups in the TCGA database. B) Analysis of AhR expression in the N0 and N1 groups in the TCGA database. C) Analysis of AhR expression in the M0 and M1 groups in the TCGA database. D) Kaplan‒Meier curves for overall survival of BCa patients with high versus low expression of AhR. E) Representative structure of 3‐IAA and AhR after the molecular docking. F) Co‐IP and Western blotting analysis revealed the interaction between endogenous 3‐IAA and AhR. G) Immunofluorescence assays of T24 and 5637 cells treated with 3‐IAA. Data are presented as mean ± SEM. *p < 0.05, **p < 0.01, ***p < 0.001, ns: not significant. AhR: aryl hydrocarbon receptor; 3‐IAA: indole‐3‐acetic acid; BCa: bladder cancer.

AhR is considered a natural ligand for tryptophan derivatives.^[^ ^20^ ^]^ Previous studies have shown that 3‐IAA can influence host immune regulation and metabolic homeostasis by activating the AhR signaling pathway.^[^ ^21^ ^]^ Moreover, Tryptophan‐derived microbial metabolites have been shown to suppress anti‐tumor immune responses by activating the AhR in tumor‐associated macrophages.^[^ ^22^ ^]^ Given that 3‐IAA is a member of this class of ligands, we hypothesized that its effects in bladder cancer are mediated via AhR signaling. To explore the relationship between the expression of AhR and the clinical features of bladder cancer, we analyzed data from the TCGA dataset to assess AhR expression across different clinical stages. Compared with the advanced T3/T4, N1, and M1 stages, AhR expression was significantly higher in early‐stage disease (**Figure** **5**A–C). Furthermore, patients with high AhR expression had significantly longer overall survival (Figure 5D). To verify whether the stability of AhR is regulated by 3‐IAA, we performed the molecular docking, immunoprecipitation (IP) and immunofluorescence (IF) experiments. The predicted binding complex yielded a docking score of −5.6 kcal mol^−1^, indicating a potentially stable binding interaction (Figure 5E). Based on the results of IP and IF experiments, 3‐IAA was shown to bind to AhR, with co‐precipitation observed in both T24 and 5637 cells. Both proteins were primarily localized in the cytoplasm (Figure 5F,G). Overall, these results suggest that AhR may serve as a biomarker for poor prognosis in bladder cancer, and that 3‐IAA may modulate AhR signaling by directly binding to the receptor, thereby regulating cellular transcriptional activity and physiological responses.

### 2.6 Knockdown of AhR can Block the Tumor‐Suppressive Effect of 3‐IAA

**Figure 6:**
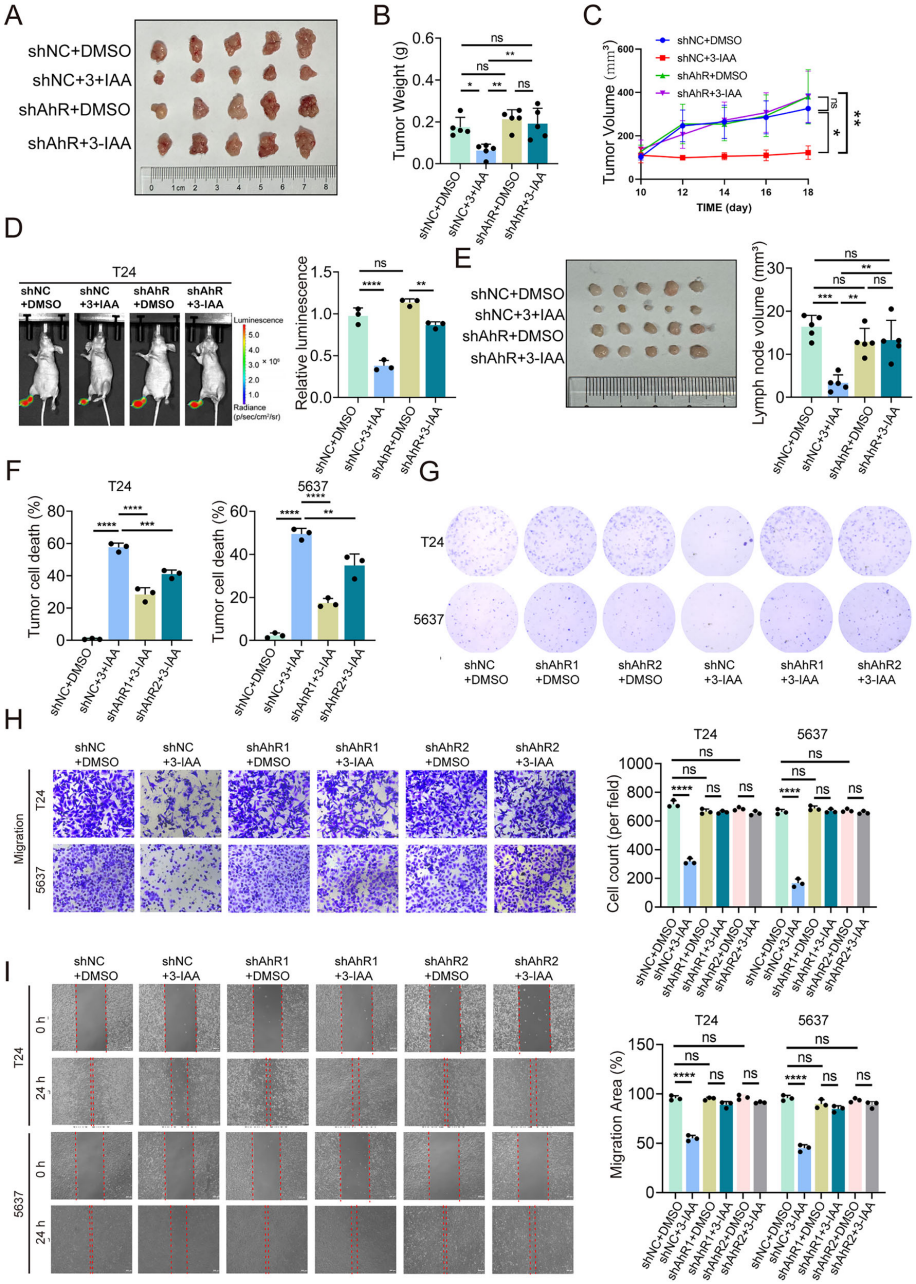
Knockdown of AhR can block the tumor‐suppressive effect of 3‐IAA. A) Images of bladder tumors, B,C) tumor volume growth curves and statistical charts of tumor weights in the mice. (n = 5) D) Representative bioluminescence images. (n = 3) E) The popliteal LN metastasis model and histogram analysis of metastatic popliteal LNs and their volumes in models established by different experimental groups. (n = 5) F) Proliferation of T24 and 5637 cells with AhR knockdown, treated with 3‐IAA was evaluated using CCK‐8 assays. G) Colony formation ability of T24 and 5637 cells under various conditions, including AhR knockdown, 3‐IAA treatment, and their combination. H) Representative images of migration assays and a histogram showing the number of migrating in T24 and 5637 cells under various conditions, including AhR knockdown, 3‐IAA treatment, and their combination. I) Representative images of wound healing assays and a histogram showing the cell migration distance in T24 and 5637 cells under various conditions, including AhR knockdown, 3‐IAA treatment, and their combination. Data are presented as mean ± SEM. *p < 0.05, **p < 0.01, ***p < 0.001, ns: not significant. 3‐IAA: indole‐3‐acetic acid; LN: lymph node; AhR: aryl hydrocarbon receptor.

We next investigated whether the tumor‐suppressive effect of 3‐IAA is dependent on AhR. To this end, we first evaluated the tumor‐suppressive role of AhR in vivo. AhR expression was knocked down in MB49 cells by shRNA transfection (Figure S4A, Supporting Information), and then subcutaneously implanted them into C57BL/6 mice. Knockdown of AhR significantly attenuated the tumor‐suppressive effect of 3‐IAA on tumor growth (**Figure** **6**A–C). Knockdown of AhR significantly reversed the reduction in Ki‐67 expression induced by 3‐IAA (Figure S4D, Supporting Information). Stable AhR‐knockdown T24 and 5637 cell lines were subsequently generated via lentiviral transduction (Figure S4B,C, Supporting Information). AhR knockdown markedly reduced the lymph node metastatic potential of T24 cells, as evidenced by decreased bioluminescence intensity (Figure 6D). Consistent results were observed in the popliteal LNs of the mice (Figure 6E). In vitro experiments revealed that AhR knockdown attenuated the anti‐proliferative and anti‐clonogenic effects of 3‐IAA on bladder cancer cells. (Figure 6F,G). Similar results were also observed in wound healing and transwell migration assays (Figure 6H,I). In addition, treatment with an AhR inhibitor (10 µM) largely reversed the anti‐proliferative and antimigratory effects mediated by 3‐IAA in bladder cancer cells (Figure S5A–G, Supporting Information). Collectively, these results indicate that 3‐IAA contributes to the tumor‐suppressive effect by activating AhR.

### 2.7 3‐IAA Activates AhR to Regulate FASN Transcription and Downregulation

**Figure 7:**
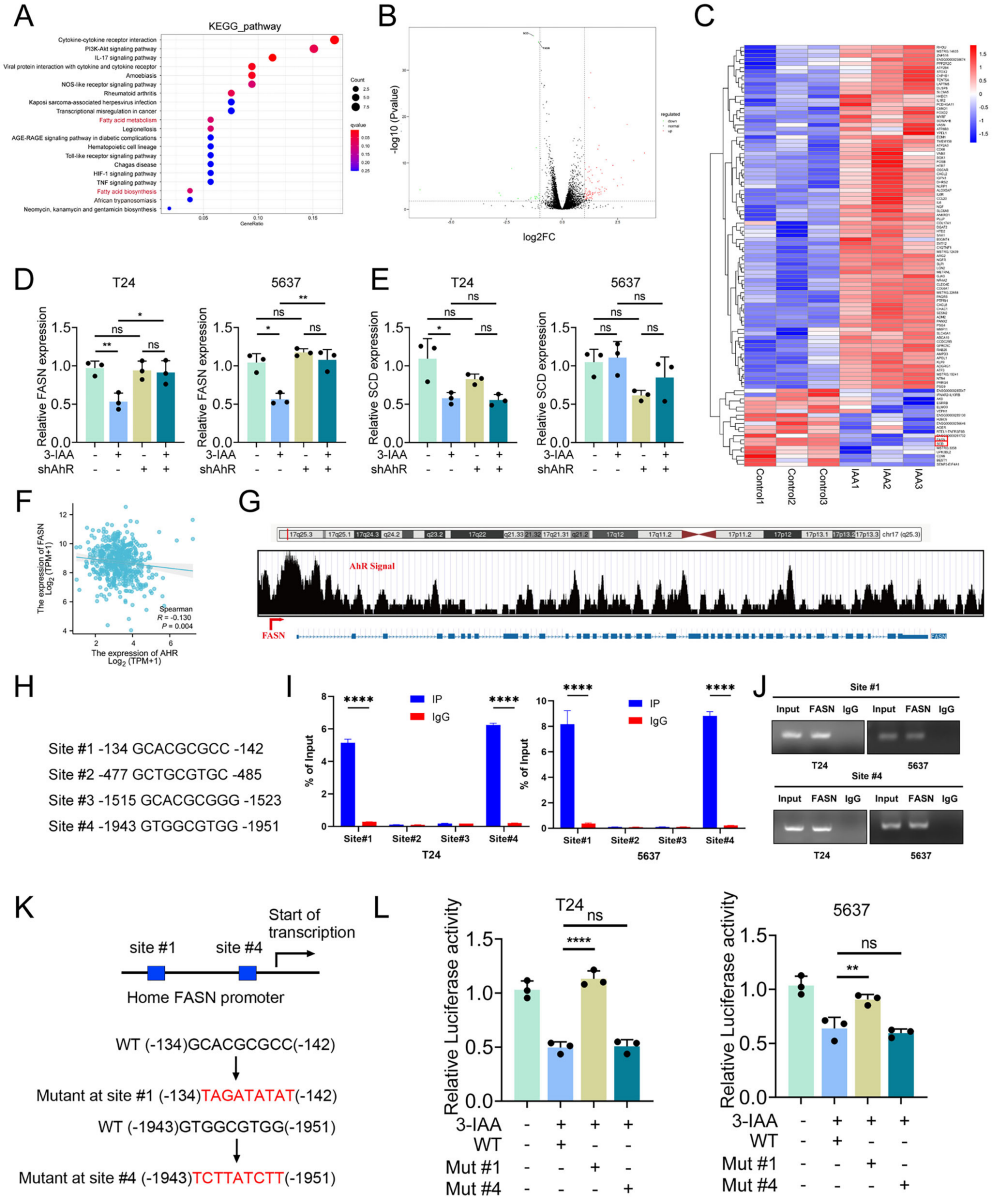
3‐IAA activates AhR to regulate FASN transcription and downregulation. A) KEGG pathway enrichment analysis of the DEGs between the 3‐IAA group and the NC group. B) Volcano plot of DEG mRNA expression. (*p* < 0.05, |log2 FC|≥1). C) Heatmap of the differential gene expression. D,E) The relative mRNA expression of FASN and SCD in T24 and 5637 cells under AhR knockdown, 3‐IAA treatment, and their combination. F) Pearson correlation analysis of the levels of AhR and FASN. G) The UCSC genome bioinformatics site showed enrichment of AhR in the promoter of FASN. H) WT sequences at binding sites 1 through 4. I,J) ChIP‒qPCR and DNA electrophoresis analyses of the binding of AhR to the FASN promoter. K) WT and Mut variants at sites 1 and 4. L) A luciferase reporter assay was used to examine the luciferase activity of WT and Mut site 1/4 after treated with 3‐IAA. Data are presented as mean ± SEM. *p < 0.05, **p < 0.01, ***p < 0.001, ns: not significant. 3‐IAA: indole‐3‐acetic acid; AhR: aryl hydrocarbon receptor; FASN: fatty acid synthase; KEGG: Kyoto Encyclopedia of Genes and Genomes; DEGs: Differentially Expressed Genes; WT: wild‐type; Mut:mutant‐type.

To explore the potential mechanism of 3‐IAA in bladder cancer, we performed RNA‐Seq analysis. KEGG pathway analysis indicated that 3‐IAA may be involved in the regulation of fatty acid metabolism and biosynthesis (**Figure** **7**A). Gene set enrichment analysis (GESA) was performed and revealed enrichment of gene signatures associated with fatty acid metabolism and biosynthesis. (Figure S6A,B, Supporting Information). To identify DEGs, we visualized the RNA‐Seq data using a volcano plot and a heatmap. Comparison of 3‐IAA‐treated and control T24 cells revealed 980 downregulated and 916 upregulated genes (Figure 7B). Furthermore, expression of the key genes FASN and stearoyl‐CoA desaturase(SCD) was significantly reduced in the 3‐IAA treatment group, both of which are associated with fatty acid metabolism and biosynthesis (Figure 7C). It has been reported that activation of the AhR receptor is significantly linked to fatty acid metabolism and biosynthesis. We therefore hypothesize that 3‐IAA‐induced activation of AhR may transcriptionally regulate the expression of FASN and SCD.

Our results showed that 3‐IAA treatment suppressed the expression of both FASN and SCD, whereas AhR knockdown restored FASN expression but had no significant effect on SCD. (Figure 7D,E).Treatment with an AhR inhibitor also reversed the downregulation of FASN expression induced by 3‐IAA (Figure S6C, Supporting Information). According to the TCGA database, FASN gene expression is strongly negatively correlated with the expression of AhR (Figure 7F). Furthermore, compared with other cancers, FASN is highly expressed in bladder cancer patients (Figure S6D, Supporting Information). Patients with high FASN expression have significantly poorer overall survival (Figure S6E, Supporting Information). Compared with patients with low‐grade tumors and those at M0 and N0 stages, patients with high‐grade tumors, as well as those at M1 and N1 stages, exhibited higher FASN expression. (Figure S6F–H, Supporting Information). The regulatory relationship between AhR and FASN was further supported by data analysis showing the presence of AhR binding signals in the FASN promoter region (Figure 7G). Next, we extracted the nucleotide sequence 2500 bp upstream of the FASN promoter and predicted potential AhR binding sites. Four canonical AhR binding sites with high predicted binding scores were identified in the FASN promoter region (Figure 7H) and subsequently validated by ChIP assays. The results of the ChIP assays confirmed the direct binding of AhR to the FASN promoter at sites 1 and 4 (Figure 7I,J). We next introduced mutations at sites 1 and 4 of the human FASN promoter sequence and constructed luciferase reporter constructs driven by either the wild‐type or mutated promoters (Figure 7K). The results indicated that 3‐IAA inhibited both the wild‐type and site 4 mutant promoters, but had no effect on the site 1 mutant promoter (Figure 7L). In conclusion, these findings indicate that 3‐IAA activates AhR to regulate FASN transcription and downregulation.

### 2.8 3‐IAA/AhR‐Mediated Downregulation of FASN Alters MUFA/PUFA Ratio to Regulate Ferroptosis In Bladder Cancer

**Figure 8:**
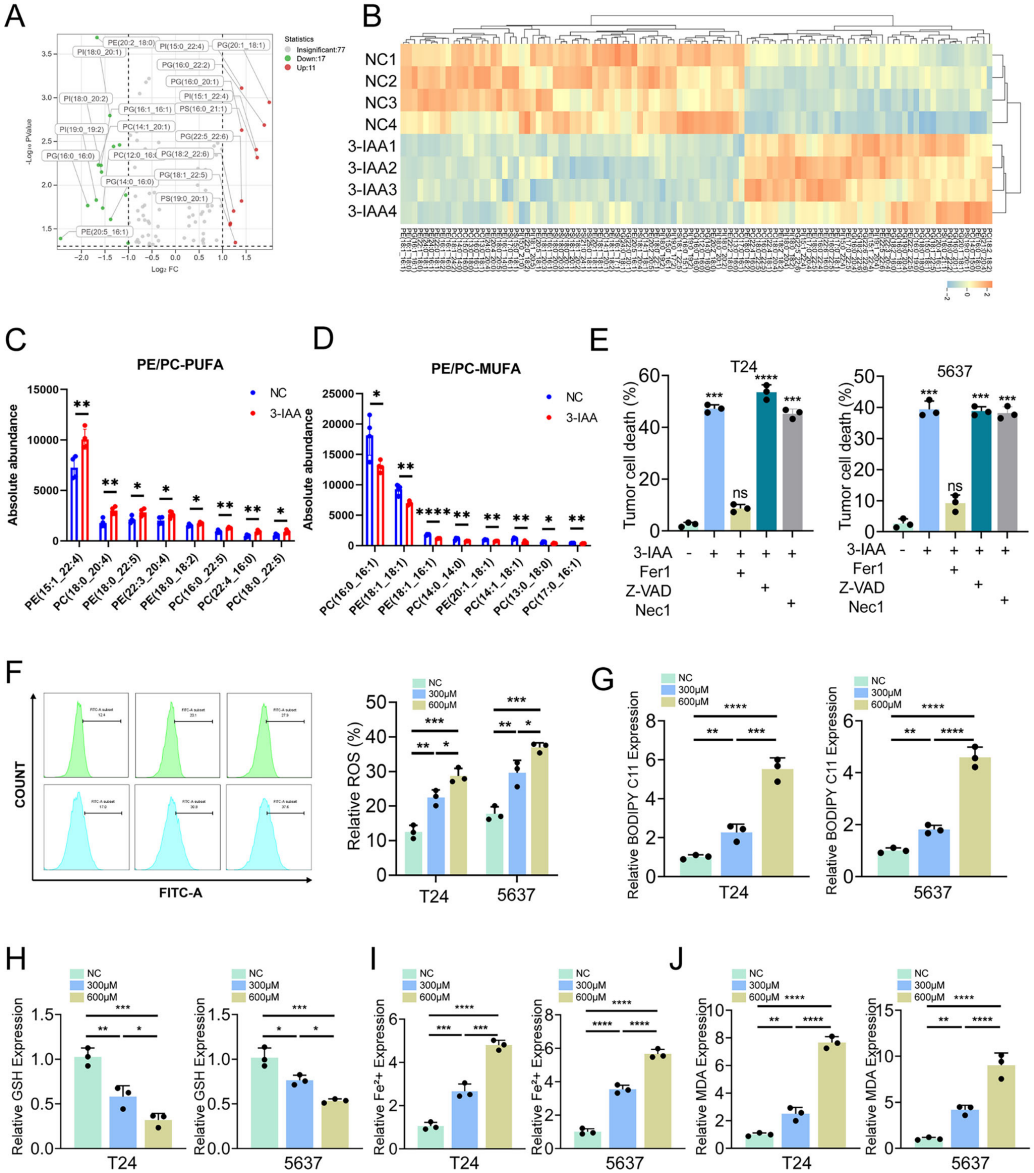
IAA/AhR‐mediated downregulation of FASN alters MUFA/PUFA ratio to regulate ferroptosis in bladder cancer. A) Volcano plots illustrating the differentially abundant metabolites between the 3‐IAA group and the NC group. (*p* < 0.05, |log2 FC|≥2). B) Heatmap of the differential PC, PE, PI, PG, and PS. C,D) The absolute abundance of PE/PC‐PUFA and PE/PC‐MUFA. E) Proliferation of T24 and 5637 cells treated with ferrostatin‐1 (5 µM, Fer1), necrostatin‐1 (10 µM, Nec1), or Z‐VAD (10 µM). F,G) ROS levels and BODIPY C11 levels were examined in T24 and 5637 cells treated with different concentrations of 3‐IAA (300 and 600 µM) or DMSO. H) GSH, I) Fe^2+^, and J) MDA levels were examined in T24 and 5637 cells treated with different concentrations of 3‐IAA (300 µM and 600 µM) or DMSO. Data are presented as mean ± SEM. *p < 0.05, **p < 0.01, ***p < 0.001, ns: not significant. 3‐IAA: indole‐3‐acetic acid; PUFA: polyunsaturated fatty acid; MUFAs: monounsaturated fatty acid; PE: phosphatidylethanolamine; PC phosphatidylcholine; PI: phosphatidylinositol; PG: phosphatidylglycerol; PS: phosphatidylserine; GSH: glutathione; MDA: malondialdehyde.

We demonstrated that 3‐IAA binding to AhR downregulates the transcription of FASN. FASN plays a key role in fatty acid synthesis, particularly in maintaining the balance between MUFAs and PUFAs, thereby indirectly influencing ferroptosis.^[^ ^17^ ^]^ High levels of FASN expression can inhibit PUFA synthesis, leading to reduced ferroptosis sensitivity. To investigate whether FASN regulates ferroptosis by mediating membrane phospholipid metabolism in bladder cancer cells, targeted lipidomic analysis was performed. Among the five major classes of membrane phospholipids (PE, PC, PI, PG and PS), the levels of MUFA‐containing species were significantly decreased, whereas PUFA‐containing species were significantly increased (**Figure** **8**A). Moreover, 3‐IAA treatment selectively increased the accumulation of polyunsaturated phosphatidylcholine (PC‐PUFAs) and phosphatidylethanolamine (PE‐PUFAs), while significantly reducing the levels of monounsaturated PC (PC‐MUFAs) and PE (PE‐MUFAs) (Figure 8B–D). We also found that 3‐IAA upregulated the expression of FADS1 and FADS2 (Figure S7A,B, Supporting Information), two key desaturase enzymes involved in the biosynthesis of PUFAs. These results indicated that downregulation of FASN enhances the incorporation of PUFAs into PC or PE, thereby competitively reducing the levels of PE/PC‐MUFAs and PC/PE‐SFAs, ultimately increasing cellular sensitivity to ferroptosis.

To further explore the involvement of 3‐IAA in ferroptosis, cells were treated with 3‐IAA alone or in combination with ferrostatin‐1 (5 µM, Fer1), necrostatin‐1 (10 µM, Nec1), or Z‐VAD (10 µM), which are specific inhibitors of ferroptosis, necrosis, and apoptosis, respectively. Compared with the 3‐IAA treatment group, Fer‐1 significantly restored the proliferation of T24 and 5637 cells, suggesting that 3‐IAA specifically regulates ferroptosis (Figure 8E). Moreover, 3‐IAA induced a dose‐dependent accumulation of ROS and lipid peroxidation (Figure 8F,G). Oral administration of 3‐IAA increased the expression of 4‐hydroxynonenal (4‐HNE) in tumor tissues, an effect that was reversed upon AhR knockdown (Figure S7C, Supporting Information). In addition, 3‐IAA treatment induced a dose‐dependent depletion of glutathione (GSH), a key regulator of ferroptosis (Figure 8H). As expected, both intracellular iron ion and malondialdehyde (MDA) levels increased in a dose‐dependent manner (Figure 8I,J). Subsequently, we constructed a FASN overexpression cells to rescue the downregulation of FASN expression induced by 3‐IAA (Figure S7D, Supporting Information). FASN overexpression reduced the accumulation of 3‐IAA‐induced ROS and lipid peroxides (Figure S7E,F, Supporting Information). FASN overexpression reduced the accumulation of 3‐IAA‐induced MDA and Fe^2+^, while increasing GSH levels (Figure S7G–I, Supporting Information). Taken together, these findings demonstrate that 3‐IAA binds to AhR and downregulates FASN transcription, thereby regulating ferroptosis in bladder cancer by disrupting the ratio of MUFAs to PUFAs.

## 3 Discussion

Recent studies have demonstrated that the gut microbiota not only helps prevent intestinal tumor development but also has protective effects on the occurrence of extraintestinal cancers.^[^ ^23^, ^24^ ^]^ Previous studies have extensively explored the relationship between the urinary microbiota and bladder cancer.^[^ ^25^ ^]^ However, the relationship between the gut microbiota and bladder tumors is still unclear, and the underlying mechanisms are not well understood. In this study, we analyzed 16S rDNA sequencing data from 50 bladder cancer patients and 22 healthy controls, and observed significant differences in the gut microbiota composition between the BC group and the control group. Through LEfSe and ROC curve analyses, *P. distasonis* was identified as a potential biomarker associated with favorable prognosis in bladder cancer patients.

Notably, *P. distasonis* has been reported as a potential next‐generation probiotic.^[^ ^26^ ^]^ *P. distasonis* plays a crucial role in human disease and health. For example, *P. distasonis* has anti‐inflammatory effects both in vivo and in vitro, and can restore the intestinal epithelial barrier.^[^ ^27^ ^]^ Wang et al.^[^ ^28^ ^]^ demonstrated that *P. distasonis* alleviates obesity, hyperglycemia, and hepatic steatosis in mice fed a high‐fat diet by producing succinate. In colorectal tumors, *P. distasonis* has been shown to inhibit signaling via TLR4, MYD88, and Akt, and to promote apoptosis, exhibiting both anti‐inflammatory and anticancer effects.^[^ ^29^ ^]^ However, the protective role of *P. distasonis* in bladder tumors has not yet been demonstrated. Hence, we provide preliminary evidence that orally administered live *P. distasonis* inhibits tumor growth in C57BL/6 mice subcutaneously inoculated with MB49 cells and inhibits lymph node metastasis in nude mice, suggesting that its tumor‐suppressive effects rely on its biological activity. We subsequently demonstrated in vitro that *P. distasonis* culture medium significantly inhibits the proliferation and migration of bladder cancer cells. Importantly, it had no effect on the proliferation of bladder epithelial cells. These findings suggest that the tumor‐suppressive effect of *P. distasonis* is primarily attributed to its metabolites, and the specific mechanism is worth further investigation.

The gut microbiota can protect against tumorigenesis through direct interactions or via the production of metabolites. Previous studies have shown that ILA derived from *Lactobacillus plantarum* enhances IL‐12 production by modulating histone modifications and chromatin accessibility in dendritic cells, thereby activating CD8^+^ T cells to suppress CRC development.^[^ ^30^ ^]^ In addition, the short‐chain fatty acid butyrate, produced by *Roseburia intestinalis*, can inhibit the development of colorectal cancer by activating cytotoxic CD8^+^ T cells.^[^ ^31^ ^]^ We identified 3‐IAA as a key metabolite produced by *P. distasonis* through untargeted liquid chromatography‒tandem mass spectrometry (LC‒MS/MS). 3‐IAA is an exclusively microbiota‐derived metabolite, and research has shown that modulating its production by the gut microbiota or its downstream targets can enhance the response to chemotherapy.^[^ ^32^ ^]^ Another study revealed that 3‐IAA ‐induced activation of the TLR4‐JNK pathway has anti‐proliferative effects on Caco‐2 cells.^[^ ^33^ ^]^ These findings suggest that the accumulation of 3‐IAA may have an inhibitory effect on tumorigenesis. Consistently, our experimental results demonstrated that 3‐IAA treatment reduces bladder tumor size in a dose‐dependent manner in C57BL/6 mice and inhibits tumor metastasis to the popliteal lymph nodes in nude mice. In vitro experiments further confirmed that 3‐IAA inhibits the proliferation and migration of bladder cancer cells in a dose‐dependent manner. These findings suggest that the use of 3‐IAA, *P. distasonis*‐derived metabolite, as an anticancer agent could be a feasible approach for treating bladder cancer.

Indole compounds, such as 3‐IAA, are recognized as natural ligands of the AhR, which translocates to the nucleus upon activation and modulates the transcription of target genes.^[^ ^20^, ^34^ ^]^ Previous studies have shown that AhR signaling plays multifaceted roles in cancer biology, exhibiting either tumor‐promoting or tumor‐suppressive effects depending on the specific ligand involved and the cellular or tissue context.^[^ ^35^, ^36^ ^]^ Given this duality and the limited understanding of AhR function in bladder cancer, we hypothesized that 3‐IAA may exert its tumor‐suppressive effects through activation of the AhR signaling pathway. We first validated that 3‐IAA can bind to AhR through IP and IF experiments. Moreover, knockdown of AhR reversed the inhibitory effects of 3‐IAA on bladder cancer both in vivo and in vitro, and similar results were observed upon treatment with an AhR inhibitor. These findings suggest that the tumor‐suppressive effects of 3‐IAA in bladder cancer are dependent on functional AhR signaling.

We then employed RNA‐Seq to identify the downstream targets of AhR. The 3‐IAA treatment group showed enrichment of gene expression changes in pathways related to fatty acid metabolism and biosynthesis, and notably, the downregulated genes included FASN and SCD. Through RT‐qPCR, ChIP, and dual‐luciferase assays, we confirmed that the activation of AhR downregulates the transcription of FASN. FASN, a central regulator of de novo lipid biosynthesis, plays a crucial role in tumorigenesis. Previous studies have shown that downregulation of FASN can inhibit tumor growth.^[^ ^37^, ^38^ ^]^ In bladder cancer, elevated expression of FASN has been associated with advanced tumor stage and poor overall survival.^[^ ^39^ ^]^ In our analysis of TCGA data, FASN expression in bladder cancer was significantly higher than that in most other cancer types, suggesting that FASN may be an important driver in the development and progression of bladder cancer.

The lipid products of FASN serve as essential precursors for phospholipid synthesis, thereby regulating the composition and function of cell membranes.^[^ ^40^ ^]^ By modulating fatty acid synthesis, particularly the balance between saturated and unsaturated fatty acids, FASN can affect cellular susceptibility to ferroptosis.^[^ ^41^ ^]^ We therefore performed targeted lipidomics to investigate changes in membrane phospholipids in cells treated with 3‐IAA. The results indicated that the 3‐IAA‐induced downregulation of FASN indirectly increased the levels of PC/PE‐PUFAs and decreased the levels of PC/PE‐MUFAs in membrane phospholipids. In addition, 3‐IAA treatment increased the levels of ROS, free iron, and MDA in bladder cancer cells, while decreasing the levels of GSH. This findings suggest that 3‐IAA induces ferroptosis by binding to AhR, thereby downregulating FASN transcription and mediating phospholipid remodeling. However, the precise mechanisms underlying MUFA regulation under these conditions remain to be fully elucidated.

Fatty acid desaturases catalyze the introduction of double bonds into fatty acyl chains, thereby modulating cellular MUFA and PUFA levels. Among these enzymes, SCD, a Δ9‐desaturase, catalyzes the conversion of saturated palmitic acid and stearic acid into the monounsaturated fatty acids palmitoleic acid and oleic acid.^[^ ^42^ ^]^ Our RNA‐seq data indicated that SCD expression was downregulated alongside FASN upon 3‐IAA treatment. However, SCD expression was not restored upon AhR knockdown, suggesting that its regulation may be independent of AhR activation. Interestingly, previous studies in hepatic cells have shown that activation of AhR can upregulate SCD expression by binding to dioxin response elements (DREs) in the SCD gene promoter, thereby promoting MUFA production.^[^ ^43^ ^]^ In contrast, our results suggest that AhR activation by 3‐IAA suppresses SCD expression, highlighting a potential cell type‐specific regulatory difference. In addition, FADS2, a Δ6‐desaturase, and FADS1, a Δ5‐desaturase, sequentially catalyze the desaturation of linoleic acid to produce highly unsaturated fatty acids, thereby modulating cellular PUFA levels.^[^ ^44^ ^]^ In our study, we found that activation of AhR by 3‐IAA led to increased expression of FADS1 and FADS2. The upregulation of FADS1 and FADS2 is likely to enhance the biosynthesis of long‐chain (PUFAs, thereby contributing to the accumulation of PUFA‐enriched phospholipids.^[^ ^45^ ^]^ Given the high susceptibility of PUFAs to lipid peroxidation, their accumulation may increase ferroptosis sensitivity. Further investigation is warranted to elucidate how AhR signaling regulates the expression or activity of FADS1 and FADS2 in bladder cancer, which may help uncover the mechanisms underlying the altered MUFA/PUFA ratio observed in our study.

Our study further expands the current understanding of the interplay between microbial metabolites, AhR signaling, and ferroptosis in cancer. Previous reports have paradoxically shown that certain bacterial tryptophan metabolites can protect cancer cells from ferroptosis.^[^ ^46^, ^47^ ^]^ For instance, trans‐3‐indoleacrylic acid (IDA), a tryptophan‐derived metabolite produced by *P. anaerobius*, has been shown to activate AhR and upregulate ALDH1A3, resulting in increased NADH production and inhibition of ferroptosis in tumor cells.^[^ ^11^ ^]^ In contrast, our study demonstrates that a different microbial indole metabolite, 3‐IAA, activates AhR signaling to induce ferroptosis and inhibit tumor growth. These findings underscore the context‐dependent nature of AhR signaling in cancer, whereby distinct ligands can exert opposing effects on ferroptosis. Based on current evidence, our study identifies a potential mechanistic link in bladder cancer by which a gut microbiota–derived metabolite induces ferroptotic cell death through the AhR‐FASN signaling pathway. These findings contribute to the growing understanding of microbiome‐host interactions, particularly in the context of lipid metabolism regulation and ferroptosis activation in malignant cells.

**Figure 9:**
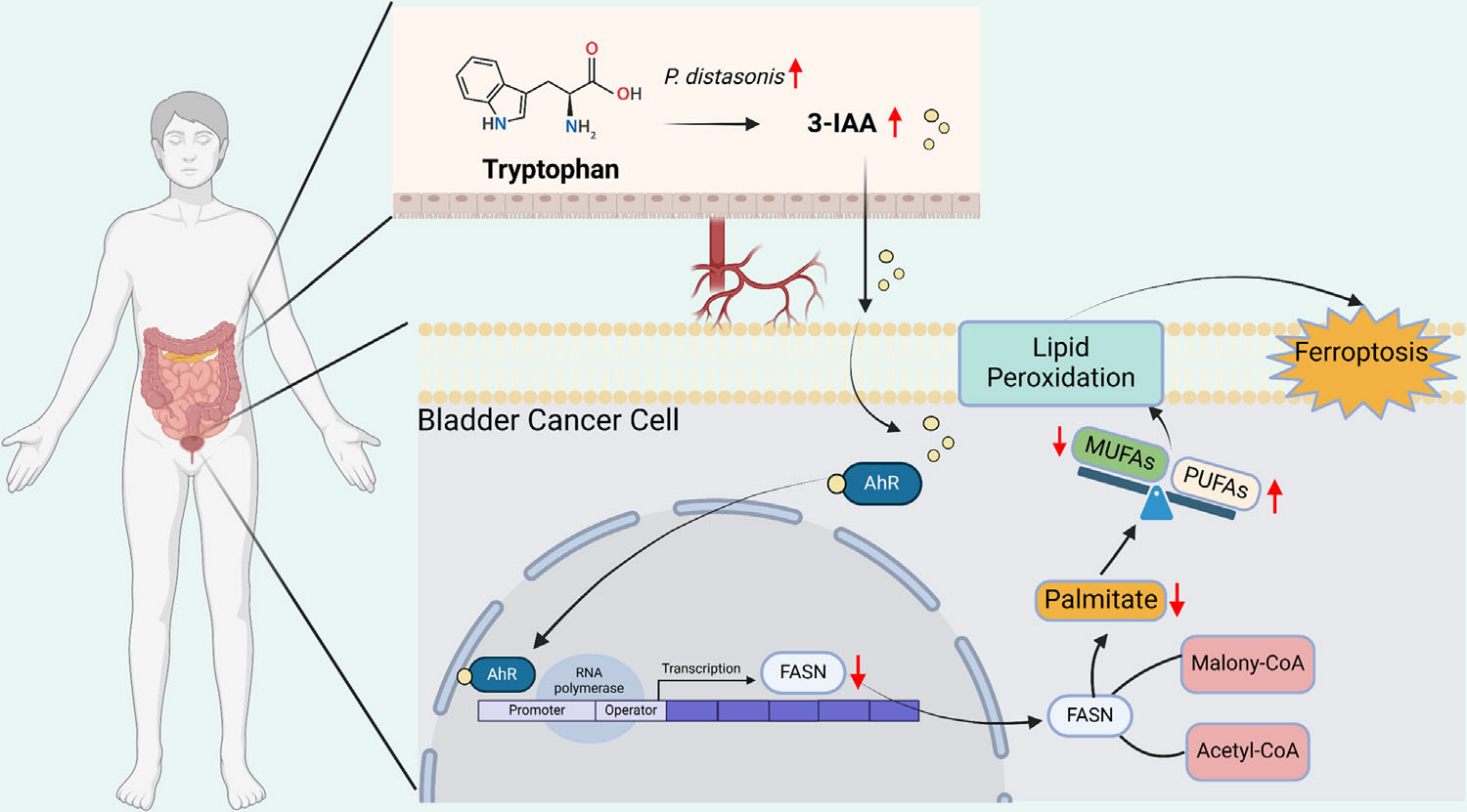
Gut *Parabacteroides distasonis*‐derived indole‐3‐acetic acid promotes phospholipid remodeling and enhances ferroptosis sensitivity via the AhR‒FASN axis in bladder cancer.

Overall, this study revealed that a high abundance of *P. distasonis* is associated with a favorable prognosis in bladder cancer patients. Moreover, in vitro and in vivo experiments confirmed that *P. distasonis* exerts its tumor‐suppressive effects by producing the metabolite 3‐IAA. Mechanistically, 3‐IAA binds to the AhR receptor on tumor cells and downregulates the transcription of FASN, thereby mediating phospholipid remodeling to increase ferroptosis sensitivity (**Figure** **9**). This discovery suggests that *P. distasonis* could be developed as a probiotic supplement for bladder cancer therapy by modulating the AhR‐FASN signaling axis. Nevertheless, further research is required to translate these findings into clinical applications. For instance, the fecal sample size in our current study was relatively limited, which may not adequately capture the full spectrum of gut microbiota alterations in bladder cancer patients. Future studies involving larger, multi‐center clinical cohorts will be essential to validate our observations and enhance their generalizability. The effective concentrations of 3‐IAA observed in experimental models should be assessed for physiological relevance, and the safety and efficacy of *P. distasonis* supplementation or 3‐IAA administration warrant comprehensive evaluation. Despite these challenges, our study demonstrated a novel therapeutic avenue that leverages the crosstalk between the gut microbiome and tumor lipid metabolism for bladder cancer treatment.

## 4 Experimental Section

### Patient Cohort and Sample Collection

All samples from bladder cancer patients were validated by postoperative pathological examination and confirmed to be urothelial carcinoma based on paraffin‐embedded tissue analysis. Cases of carcinoma in situ were carefully excluded following independent evaluations by two experienced pathologists, who additionally confirmed that none of the patients had received prior antitumor therapy. Participants, including BC patients and healthy controls, were excluded if they met any of the following criteria: (1) Use of antibiotics, proton pump inhibitors, or any other medications known to alter the gut microbiota within one month prior to fecal sample collection; or (2) A history of gastrointestinal conditions associated with gut microbiota dysbiosis, including but not limited to diarrhea, constipation, or inflammatory bowel disease (IBD). Fecal samples from both bladder cancer patients and healthy controls were collected during natural defecation using sterile collection containers and were promptly stored at −80 °C within 2 h of collection. The study protocol was approved by the Southern Medical University Ethics Committee for Research Involving Human Subjects (NFEC‐2020‐123), and all patients provided written informed consent.

### Animal Models

Animals were acquired from SPF Biotechnology Co., Ltd in Beijing, China, and maintained under specific pathogen‐free (SPF) conditions. All the mice were randomly assigned to experimental or control groups to reduce selection bias.

MB49 cells (1 × 10^6^ cells) resuspended in 200 µL of DMEM without FBS were injected subcutaneously into the right flank of the mice on day 0. Daily gavage was given until the mice were sacrificed on day 18. Blood, feces and tumors were harvested postmortem for subsequent experiments and further analysis.

For the popliteal lymph node metastasis model, BALB/c nude mice were injected by T24 (2 × 10^6^ cells) resuspended in 40 µL of serum‐free 1640 medium into their footpads. The condition of the popliteal LNs were subsequently monitored and imaged with a Vivo Imaging System (N28750). The animal experiments were approved by the Animal Ethical and Welfare Committee, Nanfang Hospital, Southern Medical University and were performed following the institutional guidelines. All research protocols were approved by the Ethics Committee of Nanfang Hospital, Southern Medical University (IACUC‐LAC‐20240513‐013).

### Bacterial Strains Culture

*P. distasonis* (obtained from the American Type Culture Collection, ATCC, USA) was cultured in brain heart infusion (BHI) broth under standard anaerobic conditions. The bacterium was incubated at 37 °C in a shaking anaerobic chamber containing 10% H2, 80% N2, and 10% CO_2_,. Prior to gavage administration, the culture was resuspended in fresh BHI broth.

When the culture of *P. distasonis* reached an optical density at 600 nm (OD_600_) of 1.0, it was centrifuged and the supernatant was filtered through a 0.22 µm pore‐size membrane to obtain the *P. distasonis* culture medium (*P.d* CM). Cells were then treated with 5% (vol/vol) *P.d* CM for 48 h.

### Cell Lines and Cell Culture

Human bladder cancer cell lines T24 and 5637 (obtained from the American Type Culture Collection, ATCC, USA) were cultured in RPMI 1640 at 37 °C and 5% CO_2_ in a humidified environment while the murine bladder cancer cell line MB49 (obtained from the American Type Culture Collection, ATCC, USA) was cultured in DMEM high glucose medium. Immortalized human ureteral epithelial cells SVHUC‐1 (obtained from the American Type Culture Collection, ATCC, USA) were cultured in F12K. Medium was supplemented with 10% fetal bovine serum (FBS) (Gibco, Indianapolis, IN, USA) and 1% penicillin/streptomycin (China).

### Cell Proliferation Assay

T24, 5637 and SVHUC‐1 cells digested and seeded into 96‐well plates at a density of 2000 cells per well. The CCK‐8 (APExBIO, USA) reagent was added after treatment according to the manufacturer's instructions, and the absorbance was measured at 450 nm.

### Wound Healing Assay

T24 and 5637 cells were seeded in 6‐well plates at a density of 500 000 cells in 3 mL of RPMI 1640 medium per well. Once the cells reached ≈90% confluence, straight scratches were created through the cell layer. The samples were gently washed twice with PBS and treated. The healing of the wounds was monitored and images were recorded using a light microscope.

### Migration Assays

T24 and 5637 (10 000 cells) were resuspended in 200 mL of FBS‐free RPMI 1640 medium and plated into the upper chamber of migrating, while 600 mL of 10% FBS‐containing RPMI 1640 cell medium or *P. d* CM was added to the lower chamber. After cultivation for 24 h at 37 °C to allow cell migration, the upper chambers were treated with 4% paraformaldehyde for 20 min to fix the cells that had migrated through the membrane. The samples were subsequently stained with 0.1% crystal violet for 10 min. Images were recorded using a light microscope.

### Untargeted Metabolomics Analysis

Liquid chromatography‒mass spectrometry (LC–MS) was employed for untargeted metabolomic analysis using an ultrahigh‐performance liquid chromatography (UHPLC) system (Agilent Technologies, Santa Clara, CA, USA). The data were subjected to preprocessing, annotation, and multivariate statistical analysis, including orthogonal partial least squares discriminant analysis (OPLS‐DA) using the ropls R package, to compare the groups. Metabolites were prioritized based on their variable importance in projection (VIP) scores: the metabolites of bacterial supernatant with a threshold of VIP>1.5 and a *p* value<0.05, whereas the serum of mice with a threshold of VIP>1 and a *p* value<0.05. An absolute fold change (FC) of at least 2 was considered significant for differentially abundant metabolites. Subsequent analysis involved KEGG pathway enrichment following the KEGG database (https://www.genome.jp/kegg/).

### ELISA

A Human ELISA Kit (Meimian Biotechnology Co., Ltd., China) was used following the manufacturer's specific protocol to measure the levels of 3‐IAA (MCE, USA). In brief, mouse serum, tumor and bacterial culture supernatants from *P. distasonis* were harvested. The treated samples were then added to wells precoated with a 3‐IAA‐specific antibody. After a 30‐min incubation and coloration at 37 °C, the absorbance of each well was recorded at 450 nm. The 3‐IAA concentration present in each well was deduced based on a standard curve.

### Cell Transfection and Lentiviral Infection

Lentiviral shRNA of shNC and shAhR were designed and synthesized by Miaoling Biology (Wuhan, China). The shRNA sequences for AhR were: shAhR (Mouse), 5′‐GGACCAGTGTAGAGCACAAATTTCAAGAGAATTTGTGCTCTACACTGGTCCT‐3′, shAhR1, 5′‐CCGGGCTCTGAATGGCTTTGTATTACTCGAGTAATACAAAGCCATTCAGAGCTTTTTG‐3′, shAhR2, 5′‐GCAGTCTGATGTCATACATCATTCAAGAGATGATGTATGACATCAGACTGCTTTTT‐3′. For FASN overexpression, the coding sequences of human FASN was cloned and inserted into the lentiviral vector pCDH. This work generated these lentiviruses in accordance with the methods outlined in the previous study.^[^ ^48^ ^]^

### RNA Sequencing and Analysis

Total RNA was extracted from the indicated cell supernatants using TRIzol (Pricella Biotechnology Co., Ltd., Wuhan, China). A KAPA RNA library prep kit was used to construct RNA sequencing libraries following the manufacturer's instructions. The Illumina NovaSeq platform was used for RNA sequencing, and the reference genome for the mice was downloaded from the GENCODE database (https://www.gencodegenes.org/mouse/).

Differentially expressed genes (DEGs) between groups were identified using the R package “edge R.” Genes with a |log2FC| >2 and a *p*‐value < 0.05 were considered DEGs. The results of the Kyoto Encyclopedia of Genes and Genomes (KEGG) enrichment analysis of DEGs were visualized using the “enrichplot” package.

### Chromatin Immunoprecipitation Assay

Chromatin immunoprecipitation (ChIP) Assay was conducted as illustrated in the former publication.^[^ ^48^ ^]^ Protein A/G magnetic beads were prepared following the instructions of MedChemExpress. The cells were harvested and washed before crosslinking with 1% formaldehyde for 10 min at room temperature, and 1.375 M glycine was added to neutralize them. The crosslinked cells were lysed on ice before centrifuged at 1350 × g for 5 min, and discard the supernatant. The samples were then sonicated using a noncontact ultrasonic disruptor. The DNA fragment size was checked by 2.5% agarose gel electrophoresis at 120 V for 15 min, with the desired size range being 200–600 bp.

Sheared DNA was divided into three aliquots for IP, IgG, and Input. The IP and IgG samples were incubated with antibody‐coupled beads, while the Input was stored at −20 °C. DNA‐protein cross‐links were reversed by incubation at 65 °C after washed, followed by treatment with RNase A and Proteinase K. DNA was purified using phenol: chloroform: isoamyl alcohol extraction and ethanol precipitation. The enrichment of FASN promoter regions was quantified by qPCR using specific primers and normalized to Input and IgG controls. The qPCR data were analyzed using the 2^‐ΔΔCt method to determine the relative enrichment of FASN promoter sequences.

### Western Blotting Analysis

After specific treatments, BCa cells were lysed with RIPA lysis buffer (Cell Signaling Technology) supplemented with a Protease/phosphatase inhibitor cocktail (New Cell & Molecular Biotech, Suzhou). Equal amounts of protein extracts were separated by sodium dodecyl sulfate‐polyacrylamide gel electrophoresis (SDS‐PAGE). Following separation, the proteins were transferred to polyvinylidene fluoride (PVDF) membranes. To prevent non‐specific binding, the PVDF membrane was blocked using a 5% bovine serum albumin (BSA) solution in TBST buffer. The blocked membrane was then incubated with a specific primary antibody overnight at 4 °C and a secondary antibody. Protein bands were visualized using the GelView 6000 Pro from BLT (China). The antibodies used in this study were listed at Table S2, Supporting Information.

### qPCR/ Real‐Time Quantitative PCR

RNA isolation and quantitative real‐time polymerase chain reaction (qPCR) were performed as previously described.^[^ ^48^ ^]^ Briefly, total RNA was extracted using TRIzol (Pricella Biotechnology Co., Ltd., Wuhan, China). cDNA was obtained from RNA reverse transcription using HiScript III RT SuperMix for qPCR (Vazyme Biotech Co., Ltd, Nanjing, China). RT‒qPCR was performed to evaluate the expression of the corresponding RNA in different cell types. The primer used in this study were listed at Table S3, Supporting Information.

### Immunofluorescence Staining

Immunofluorescence (IF) staining was performed in accordance with methodologies described.^[^ ^48^ ^]^ In brief, BCa cells were seeded onto confocal dishes and fixed using 4% paraformaldehyde, followed by pre‐hybridization with 0.5% Triton X‐100. Subsequently, cells were blocked and incubated overnight with primary antibodies at 4 °C. The specific primary antibodies in this research are detailed in Table S1, Supporting Information. Post‐incubation, the dishes underwent a thrice‐repeated washing with PBS, followed by a 1‐h incubation with secondary antibodies at 25 °C. Cells were then stained with DAPI (Beyotime, Shanghai, China) for 5 min at 25 °C for nuclear counterstaining. Imaging was performed using a confocal microscope (Zeiss, Munich, Germany).

### Targeted Lipidomics

The sample was taken out from the −80 °C refrigerator, thawed on ice and vortexed for 10 s. Mix 50 µL of the sample and 1 mL of the extraction solvent (MTBE: MeOH = 3:1, v/v) containing internal standard mixture. After whirling the mixture for 15 min, 200 µL of ultrapure water was added. Vortex for 1 min and centrifuge at 12 000 rpm for 10 min. 200 µL of the upper organic layer was collected and evaporated using a vacuum concentrator. The dry extract was dissolved in 200 µL reconstituted solution (ACN: IPA = 1:1, v/v) to LC‐MS/MS analysis. The extraction, detection, and quantitative analysis of metabolites in the samples were performed by Wuhan Metware Biotechnology Co., Ltd. (www.metware.cn);

### Immunohistochemistry

Immunohistochemistry (IHC) were performed as previously described.^[^ ^49^ ^]^ Briefly, immunohistochemistry was performed on sections of bladder cancer tissues, which had been fixed in 10% formalin and paraffin‐embedded. Antigen retrieval was conducted using sodium citrate/EDTA after dewaxing and rehydration. The sections were then incubated with the primary antibody targeting ki67 (Proteintech, 27309‐1‐AP, diluted at 1:200) or 4‐HNE (Bioss, bs‐6313R, diluted at 1:200) at 4 °C overnight. On the following day, the sections were HRP‐labeled, developed with DAB, and counterstained with hematoxylin. The resulting images were captured via microscopy and analyzed using Image J software.

### Reactive Oxygen Species Detection and Lipid Peroxidation

Manufacturer's protocol was followed to detect the intracellular ROS levels of T24 and 5637 cells using a reactive oxygen species (ROS) assay kit (Solarbio Science & Technology Co., Ltd, Beijing, China). Lipid peroxidation was assessed using the BODIPY 581/591 C11 fluorescent probe kit (Beyotime, Shanghai, China). The relative ROS levels and lipid peroxidation were detected by spectrophotometer or flow cytometry at excitation/emission wavelengths of 488/510 (traditional FITC filter set) and analyzed by GraphPad Prism 10.0 software.

### Malondialdehyde Assay

The relative MDA levels of T24 and 5637 cells were measured using an MDA activity assay kit (BC0025, Solarbio Science & Technology Co., Ltd, Beijing, China) following the manufacturer's instructions. In brief, the cells supernatant was collected and centrifuged for detecting MDA levels. And the absorbance was measured at 520 and 600 nm for further analysis.

### Reduced Glutathione Assay

The relative reduced GSH levels of T24 and 5637 cells were measured using a reduced GSH activity assay kit (BC1175, Solarbio Science & Technology Co., Ltd, Beijing, China) following the manufacturer's instructions. In brief, the cells were collected and sonicated using a non‐contact ultrasonic disruptor, followed by centrifuging for reduced GSH levels detection. And the absorbance was measured at 412 nm for further analysis.

### Colorimetric Ferrozine‐Based Assay

Colorimetric ferrozine‐based assay was performed to measure the quantitation of Fe2+ using a ferrous iron activity assay kit (ADS‐W‐QT027, Aidisheng Biological Technology Co., Ltd, Jiangsu, China) following the manufacturer's instructions. Briefly, the cells were collected and sonicated using a non‐contact ultrasonic disruptor, followed by centrifuging for Fe2+ levels detection. The absorbance was measured at 562 nm for further analysis.

### Molecular Docking Analysis

To analyze the AhR receptor‐3‐IAA interactions, a receptor‐ligand interaction analysis was performed. The 3D structure of 3‐IAA was sourced from the PubChem database and converted into PDB format utilizing PyMOL visualization software. The structure of AhR (PDB code: 4M4X) was downloaded from the Protein Data Bank(www.rcsb.org). AutoDockTool was used for docking procedure. The top five results with the highest binding energy values were chosen for further analysis.

### Statistical Analysis

GraphPad Prism 10.0 was used for statistical analysis, and the date are presented as means ± SEM. The sample size (n) for each statistical analysis is provided in the figure legends. Independent Student's *t*‐test was used to compare the difference between two groups, and multiple *t*‐tests were used to compare the difference at different timepoints or stages between two groups. The Mann–Whitney U‐test was used to analyze nonparametric data, with the significance threshold set at *p* < 0.05. While one‐way ANOVA along with Tukey's test was used for multiple group comparisons. As shown in the figure legend, significance levels are marked as *p < 0.05, **p < 0.01, and ***p < 0.001, ns indicates no significant difference.

## Conflict of Interest

The authors declare no conflict of interest.

## Supporting information



Supporting Information

## Data Availability

Research data are not shared.
