# Correction to “Gut *Parabacteroides distasonis*‐Derived Indole‐3‐Acetic Acid Promotes Phospholipid Remodeling and Enhances Ferroptosis Sensitivity via the AhR‒FASN Axis in Bladder Cancer”

**DOI:** 10.1002/advs.77485

**Published:** 2026-09-27

W. Li, W. Shangguan, W. Huang, et al. “Gut Parabacteroides distasonis‐derived Indole‐3‐Acetic Acid Promotes Phospholipid Remodeling and Enhances Ferroptosis Sensitivity via the AhR‐FASN Axis in Bladder Cancer,” *Advanced Science* 12, no. 34 (2025): 12, e04688. https://doi.org/10.1002/advs.202504688

During figure assembly, several representative images were inadvertently assigned to incorrect experimental groups due to unintentional errors in image organization. Specifically, a representative colony formation image in Figure 4I, representative bioluminescence images in Figure 6D were incorrectly assigned. These panels have now been replaced with provenance‐verified original images from the correct experimental groups. Following identification of these issues, all original image files and assembled figures were comprehensively re‐examined to verify the accuracy of image assignment. No additional image assignment errors were identified. These corrections do not affect the interpretation of the data, the reported results, or the conclusions of the study. The corrected Figures 4I and 6D are as follows:

Corrected Figure 4

FIGURE 43‐IAA derived from *P. distasonis* can exert tumor‐suppressive effects. (A) Schematic diagram of the subcutaneous tumor model established in C57BL/6 mice. (B) Images of bladder tumors, (C) statistical charts of the tumor weights, and (D) tumor volume growth curves in mice. (*n* = 5) (E) Schematic representation of the establishment of a popliteal LN metastasis model in nude mice. (F) Representative bioluminescence images. (*n* = 3) (G) The popliteal LN metastasis model and histogram analysis of metastatic popliteal LNs and their volumes in models established by different experimental groups. (*n* = 5) (H) The proliferation of T24 and 5637 cells treated with different concentrations of 3‐IAA for 24 or 48 h was evaluated using CCK‐8 assays. IC_5_₀ values are shown. (I) Colony formation of T24 and 5637 cells treated with different concentrations of 3‐IAA (300 and 600 µm) or DMSO. (J) Representative images of migration assays and a histogram showing the number of migrating in T24 and 5637 cells treated with different concentrations of 3‐IAA (300 and 600 µm) or DMSO. (K) Representative images of wound healing assays and (L) a histogram showing the cell migration distance in T24 and 5637 cells treated with different concentrations of 3‐IAA (300 and 600 µm) or DMSO. Data are presented as mean ± SEM. *p < 0.05, **p < 0.01, ***p < 0.001; ns: not significant. 3‐IAA: indole‐3‐acetic acid; LN: lymph node; IC_50:_ half maximal inhibitory concentration.

**Figure:**
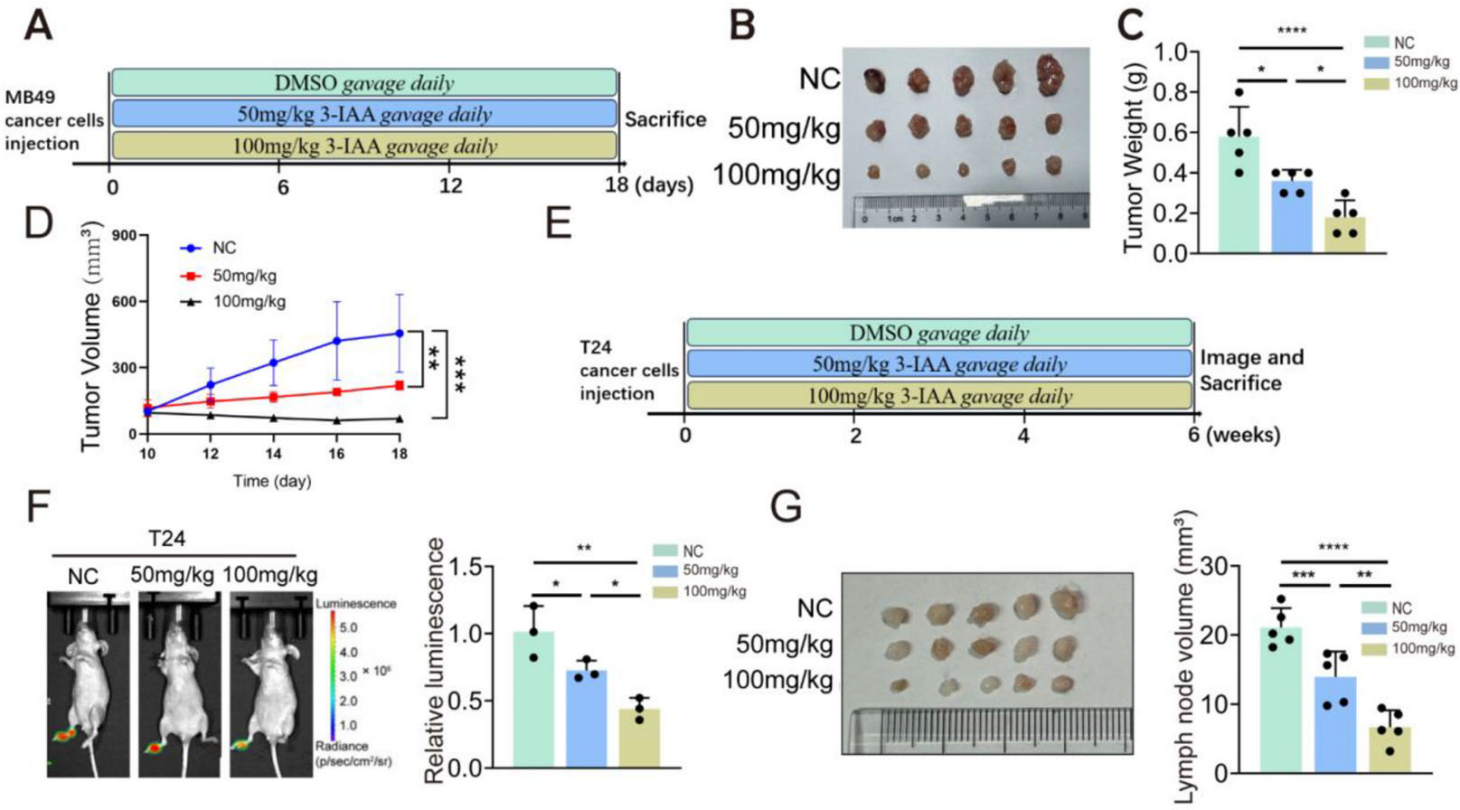

**Figure:**
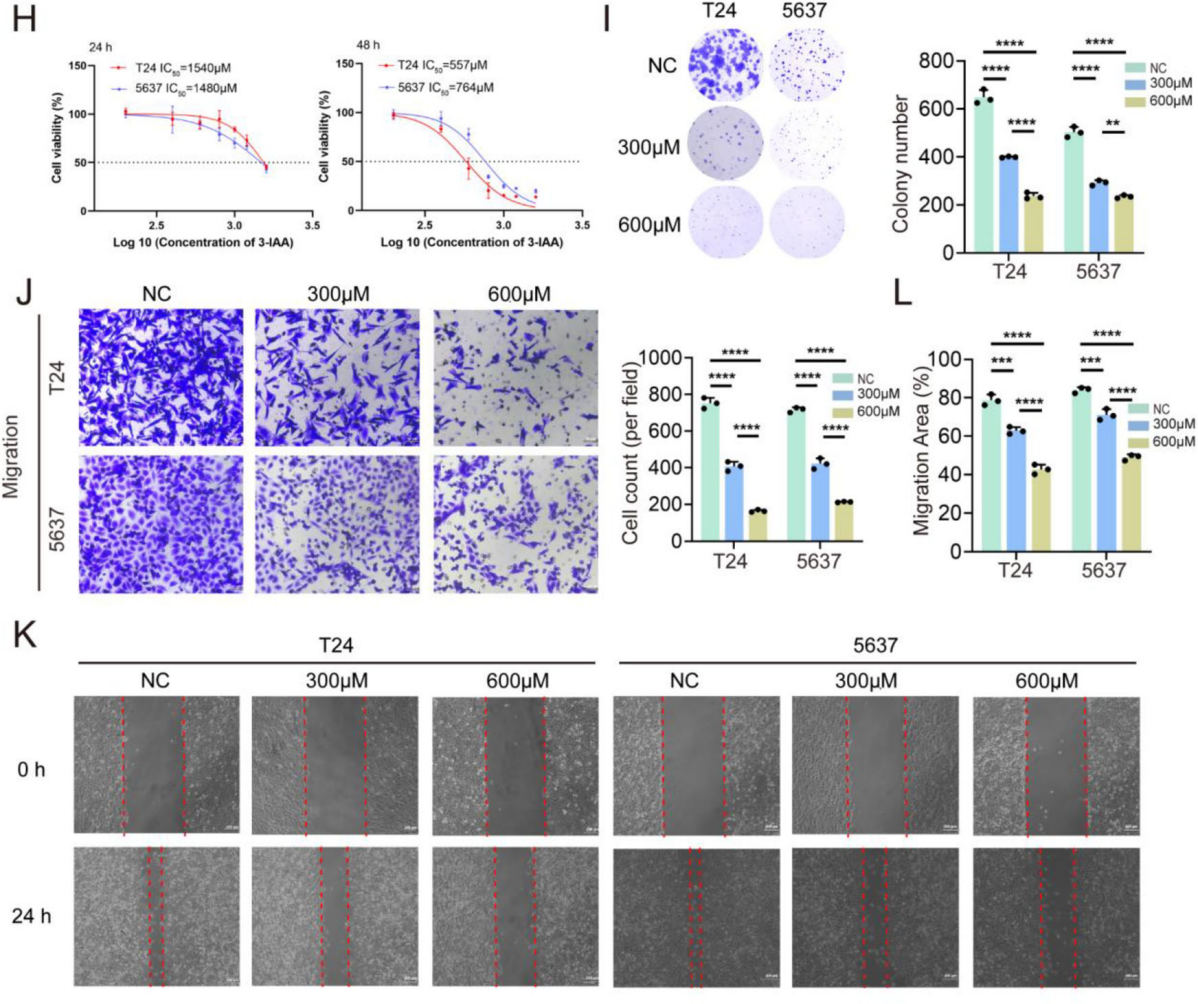

Corrected Figure 6

**FIGURE 6:**
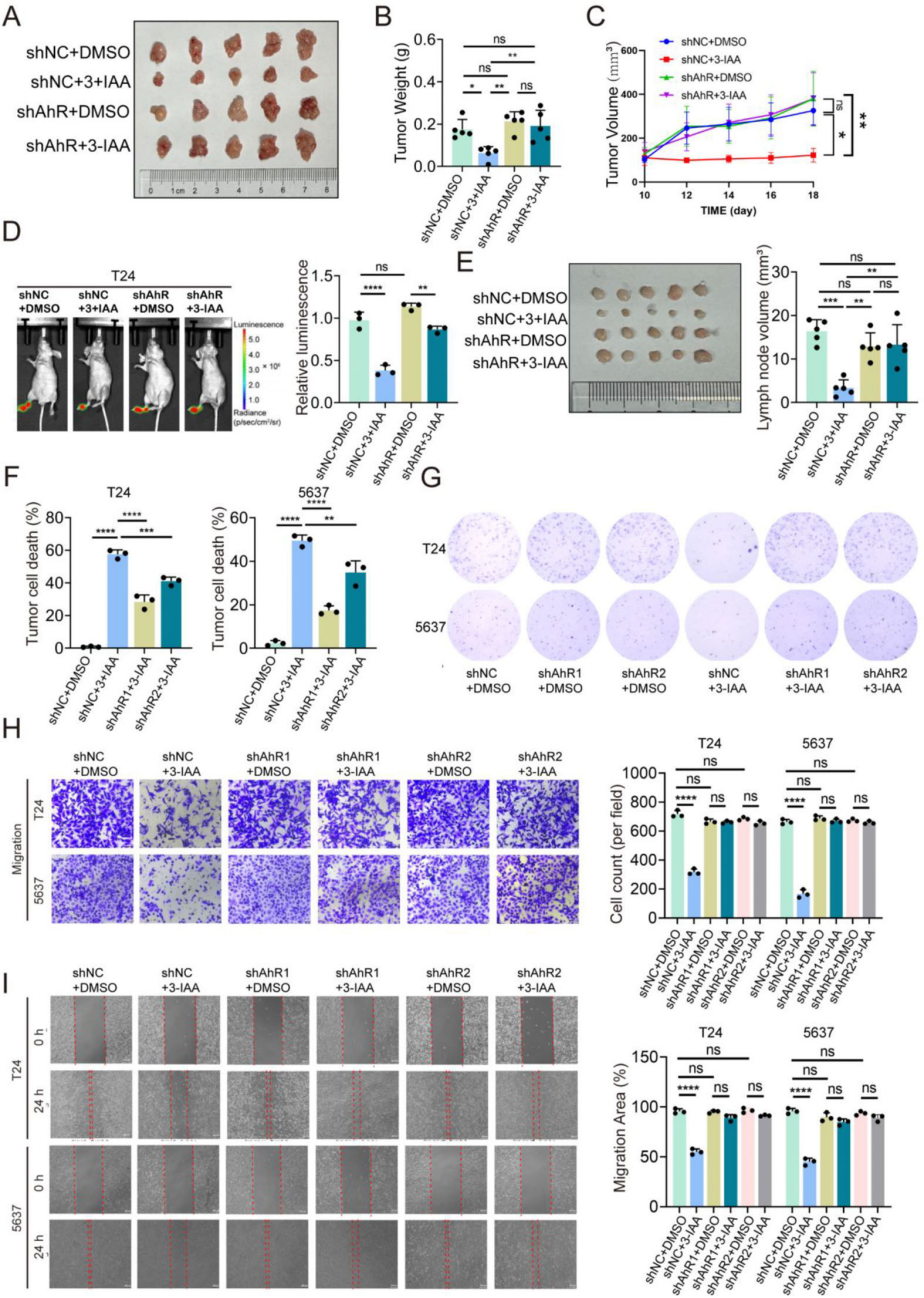
Knockdown of AhR can block the tumor‐suppressive effect of 3‐IAA. (A) Images of bladder tumors, (B, C) tumor volume growth curves and statistical charts of tumor weights in the mice. (*n* = 5) (D) Representative bioluminescence images. (*n* = 3) (E) The popliteal LN metastasis model and histogram analysis of metastatic popliteal LNs and their volumes in models established by different experimental groups. (*n* = 5) (F) Proliferation of T24 and 5637 cells with AhR knockdown, treated with 3‐IAA was evaluated using CCK‐8 assays. (G) Colony formation ability of T24 and 5637 cells under various conditions, including AhR knockdown, 3‐IAA treatment, and their combination. (H) Representative images of migration assays and a histogram showing the number of migrating in T24 and 5637 cells under various conditions, including AhR knockdown, 3‐IAA treatment, and their combination. (I) Representative images of wound healing assays and a histogram showing the cell migration distance in T24 and 5637 cells under various conditions, including AhR knockdown, 3‐IAA treatment, and their combination. Data are presented as mean ± SEM. *p < 0.05, **p < 0.01, ***p < 0.001, ns: not significant. 3‐IAA: indole‐3‐acetic acid; LN: lymph node; AhR: aryl hydrocarbon receptor.

We apologize for these errors.

