# Supplementary material for: Gut Parabacteroides distasonis‐derived Indole‐3‐Acetic Acid Promotes Phospholipid Remodeling and Enhances Ferroptosis Sensitivity via the AhR‐FASN Axis in Bladder Cancer
Source: Adv Sci (Weinh). 2025 Jun 25;12(34):e04688. doi: 10.1002/advs.202504688 (PMC12442663; doi:10.1002/advs.202504688)
Supplement: Supplementary file 1 — Supporting Information [file ADVS-12-e04688-s001.docx]

**Supplementary Table****s**

Supplementary Table S1. Baseline characteristics.

| Characteristics |  | Patients (n=40) | Low *P. distasonis* (n=24) | High *P. distasonis* (n=16) | p |
| --- | --- | --- | --- | --- | --- |
| Ages (years) | Median(range) | 64(29-85) | 64(40-85) | 63(29-85) | 0.802 |
| Gender | Male | 32(80.00%) | 16(66.67%) | 16(100.00%) | 0.01 |
|  | Female | 8(20.00%) | 8(33.33%) | 0(0.00%) |  |
| BMI | Median(range) | 23.3(17.5-28.1) | 23.1(17.5-28) | 23.6(19.3-28.1) | 0.615 |
| Grade | PUNMLP | 6(15.00%) | 1(4.17%) | 5(31.25%) | 0.003 |
|  | Low | 14(35.00%) | 7(29.17%) | 7(43.75%) |  |
|  | High | 20(50.00%) | 16(66.67%) | 4(25.00%) |  |
| pT | NMIBC | 32(80.00%) | 18(75.00%) | 14(87.50%) | 0.333 |
|  | MIBC | 8(20.00%) | 6(25.00%) | 2(12.50%) |  |
| Smoking history | Y | 23(57.50%) | 13(54.17%) | 10(62.50%) | 0.601 |
|  | N | 17(42.50%) | 11(45.83%) | 6(27.50%) |  |

Low *P. distasonis* group (*P. distasonis* expression < 0.3585275) and High *P. distasonis* group (*P. distasonis* expression > 0.3585275).

Supplementary Table S2. Antibodies used in this study

| **Application** | **Protein** | **Manufacturer** | **Cat number** | **Dilution for usage** |
| --- | --- | --- | --- | --- |
| Western blotting | AhR | Cloud-Clone | MAB354HU22 | 1:1000 |
| Western blotting | 3-IAA | Cloud-Clone | PAA737GE01 | 1:1000 |
| Western blotting | FASN | Proteintech | 10624-2-AP | 1:5000 |
| Western blotting | GAPDH | Proteintech | 10494-1-AP | 1:5000 |
| Immunohistochemistry | ki67 | Proteintech | 27309-1-AP | 1:200 |
| Immunohistochemistry | 4-HNE | Bioss | bs-6313R | 1:200 |
| Immunofluorescence | 3-IAA | Cloud-Clone | PAA737GE0 | 1:50 |
| Immunofluorescence | AhR | Cloud-Clone | MAB354HU22 | 1:50 |
| Immunoprecipitation | 3-IAA | Cloud-Clone | PAA737GE0 | 1:100 |
| Immunoprecipitation | AhR | Cloud-Clone | MAB354HU22 | 1:100 |

Manufacturer’s information: Cloud-Clone (Wuhan, Hubei, China). Proteintech (Wuhan, Hubei, China). Bioss (Beijing, China).

Supplementary Table S3. Primers used in this study

| **Primer Name** | **Sequence 5’-3’** |
| --- | --- |
| **Used in qPCR** |  |
| *s_Bacteroides fragilis* Forward | ATAGCCTTTCGAAAGRAAGAT |
| *s_Bacteroides fragilis* Reverse | CCAGTATCAACTGCAATTTTA |
| *s_Prevotella copri* Forward | CTCCAGCGCCAAGACTCAGA |
| *s_Prevotella copri* Reverse | ACCCTTGAAACTGCCACCCA |
| *s_Parabacteroides distasonis* Forward | GGACACGTCCCGCACTTTAT |
| *s_Parabacteroides distasonis* Reverse | TTCTGAGAGGAAGGTCCCCC |
| AhR Forward | TCAAATCCTTCCAAGCGGCA |
| AhR Reverse | ACAGTTATCCTGGCCTCCGT |
| FASN Forward | CTACCTGAGCATAGTGTGGAAGACGCTG |
| FASN Reverse | CATCCCACTGGTACACCTTCCCACTCAC |
| SCD Forward | AAACCTGGCTTGCTGATG |
| SCD Reverse | GGGGGCTAATGTTCTTGTCA |
| FADS1 Forward | CCTGGAAAGCAACTGGTTTGTG |
| FADS1 Reverse | GAAGGCAGACTTGTGGACATTG |
| FADS2 Forward | AAGGGAGGTAACCAGGGAGAG |
| FADS2 Rorward | CCGCTGGGACCATTTGGTAA |
| GAPDH Forward | AGGTTGTCTCCTGCGACTGCA |
| GAPDH Reverse | GTGGTCCAGGGTTTCTTACTCC |
| **Used in ChIP-qPCR** |  |
| *FASN* Site#1 Forward | CTCCTGGGTTCAAGCTATTCTC |
| *FASN* Site#1 Reverse | AGGGAAACTCCGTCTCTACTAA |
| *FASN* Site#2 Forward | CCTCACCCTGAATCAGAAGAAC |
| *FASN* Site#2 Reverse | GCTGCGTGTCCTACTTTGA |
| *FASN* Site#3 Forward | GATTGGTTGCTGTGCCG |
| *FASN* Site#3 Reverse | GATGGGAATGCTTGGGCCA |
| *FASN* Site#4 Forward | CTCCTCAGTCCCAGCCCC |
| *FASN* Site#4 Reverse | CGCCGACGCTATTTAAACCG |


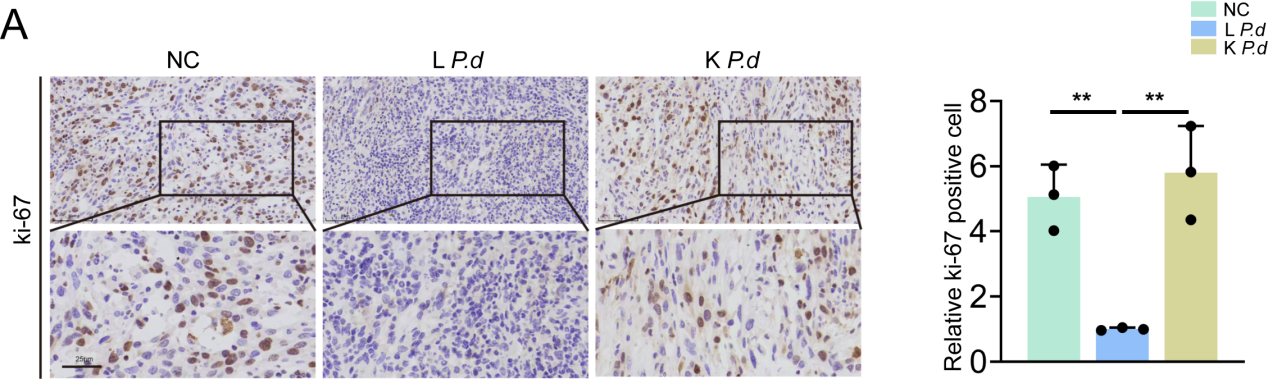


**Figure S1**

L *P.d* reduced the expression of Ki67 in tumors. (A) Immunohistochemical staining show ki67 expression in tumors. Data are represented as mean ± SEM. **P* < 0.05, ***P* < 0.01, ****P* < 0.001, ns: not significant. L *P.d*: Live *P.* *distasonis*.


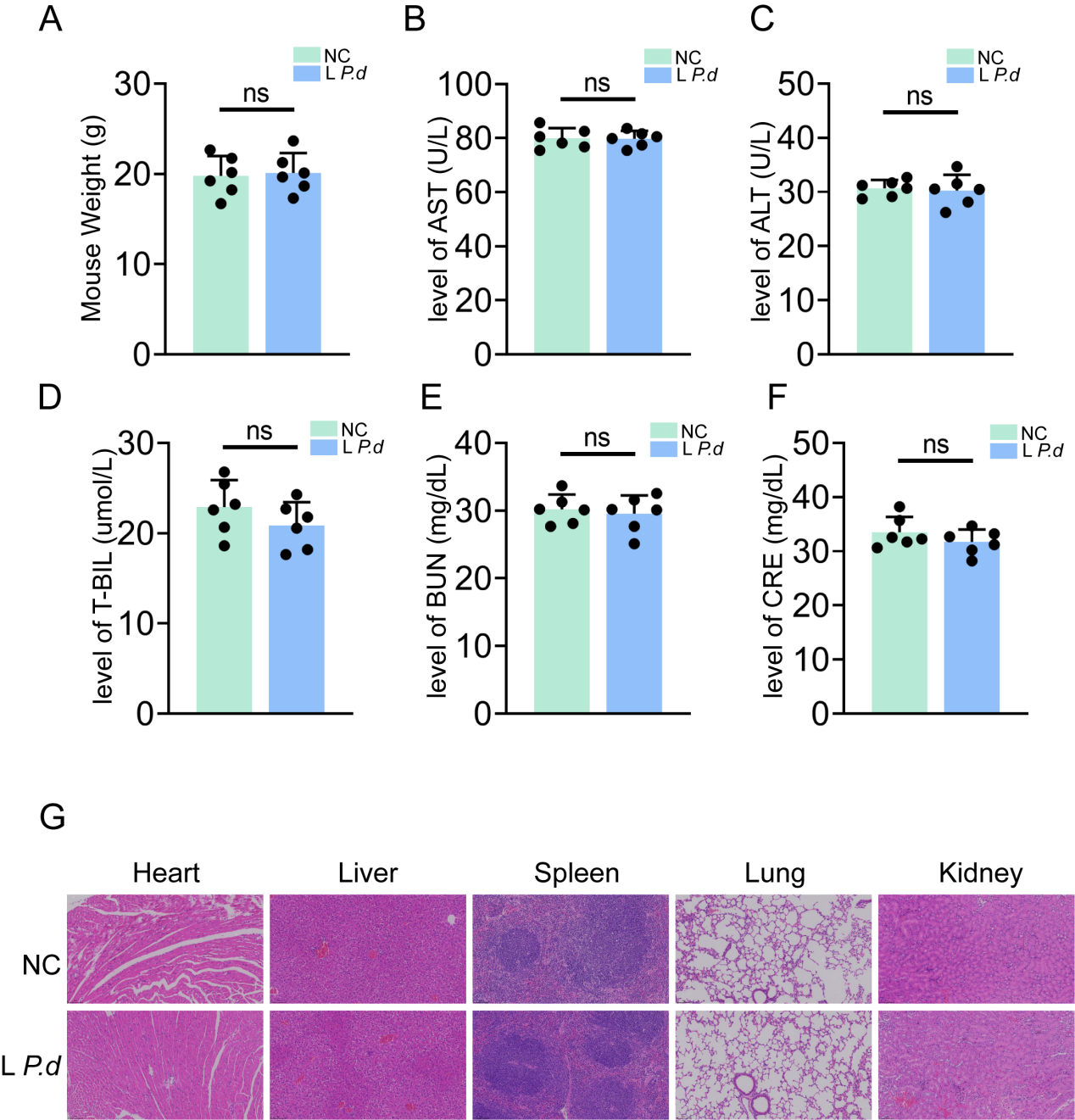


**Figure S2**

Oral administration of L *P.d* exhibited no obvious toxicity or adverse effects in mice. (A) Statistical comparison of body weights between NC and L *P.d* groups of mice. (B) Liver function in NC and L *P.d* groups was assessed by measuring serum levels of AST (B), ALT (C), and T-BIL (D). Kidney function was evaluated based on BUN (E) and CRE (F) levels. (G) Representative hematoxylin and eosin (H&E) staining images of the liver, kidney, lung, heart, and spleen from mice in the NC and L *P.d* groups. ns: not significant. AST: aspartate aminotransferase; ALT: alanine aminotransferase; T-BIL: total bilirubin; BUN: blood urea nitrogen; CRE: serum creatinine.

**
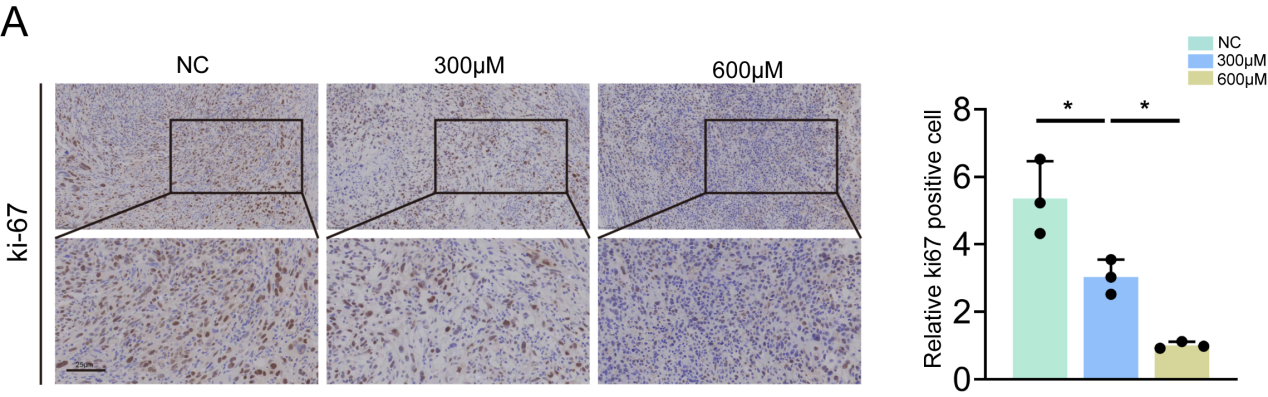
**

**Figure S3**

3-IAA reduced the expression of Ki67 in tumors. (A) Immunohistochemical staining show ki67 expression in tumors. Data are represented as mean ± SEM. **P* < 0.05, ***P* < 0.01, ****P* < 0.001, ns: not significant. L *P.d*: Live *P.* *distasonis*.

**
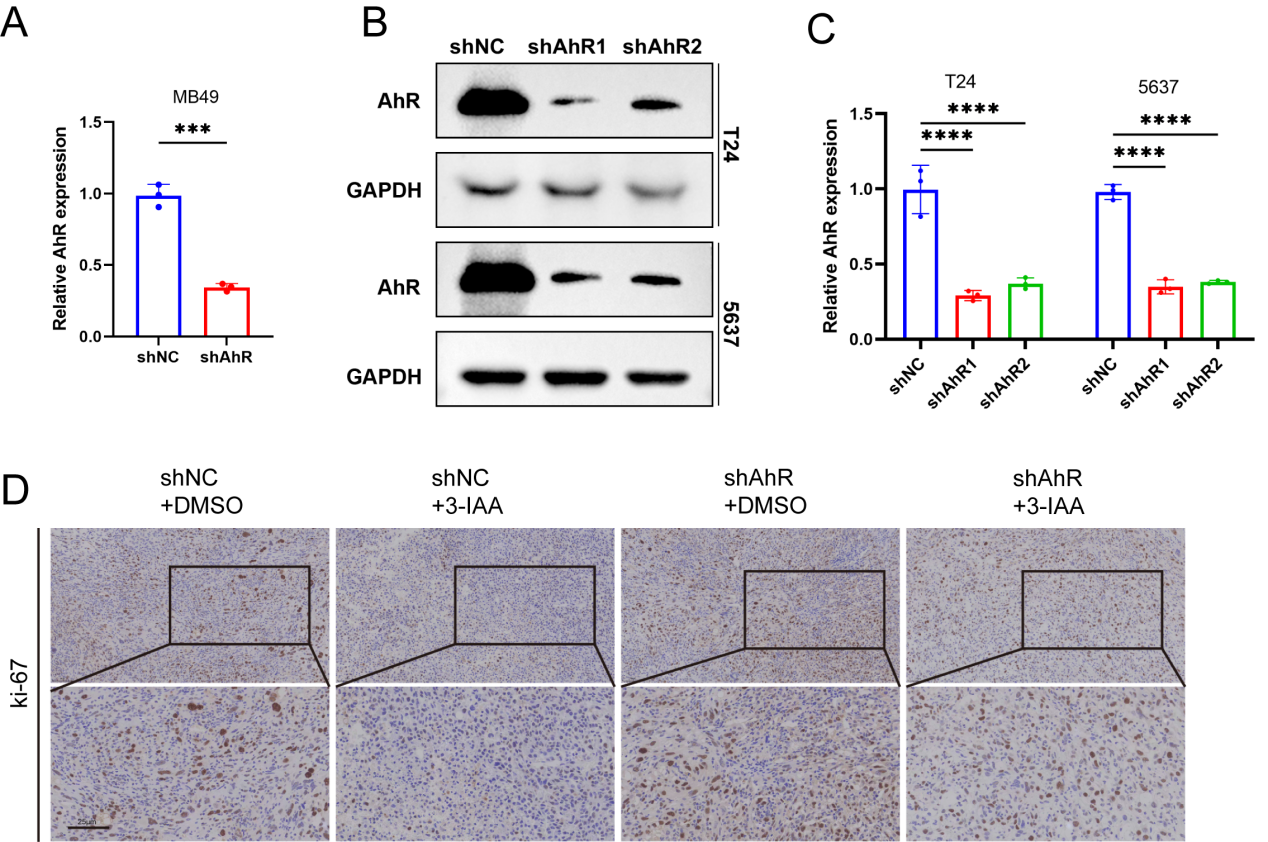
**

**Figure S4**

Construction of AhR knockdown cell lines. (A) RT-qPCR analysis of AhR expression in AhR knockdown MB49 cells. (B) Immunoblot analysis of AhR expression in AhR knockdown T24 and 5637 cells. (C) RT-qPCR analysis of AhR expression in AhR knockdown T24 and 5637 cells. (D) Immunohistochemical staining show ki67 expression in tumors. Data are represented as mean ± SEM. **P* < 0.05, ***P* < 0.01, ****P* < 0.001, ns: not significant. AhR: Aryl hydrocarbon receptor.


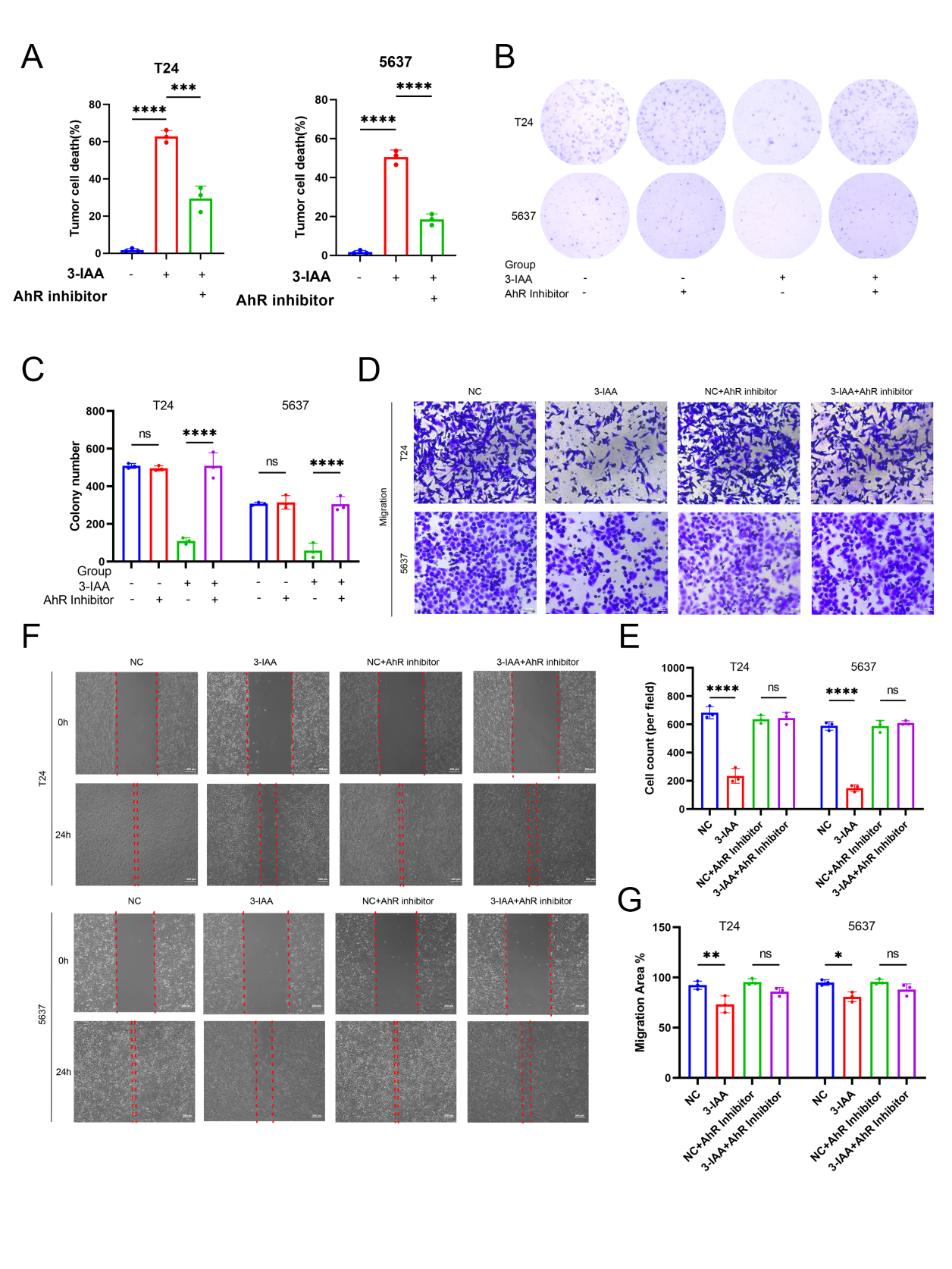


**Figure S5**

AhR inhibitors can block the tumor-suppressive effects of 3-IAA. (A) Proliferation of T24 and 5637 cells treated with an AhR inhibitor (10 µM). (B-C) Colony formation ability of T24 and 5637 cells treated with the AhR inhibitor. (D-E) Representative images of migration assays and a histogram showing the number of migrating in T24 and 5637 cells treated with the AhR inhibitor. (F-G) Representative images of wound healing assays and a histogram show cell migration distance in T24 and 5637 cells treated with AhR inhibitor. Data are represented as mean ± SEM. **P* < 0.05, ***P* < 0.01, ****P* < 0.001, ns: not significant. AhR: Aryl hydrocarbon receptor; 3-IAA: indole-3-acetic acid.

**
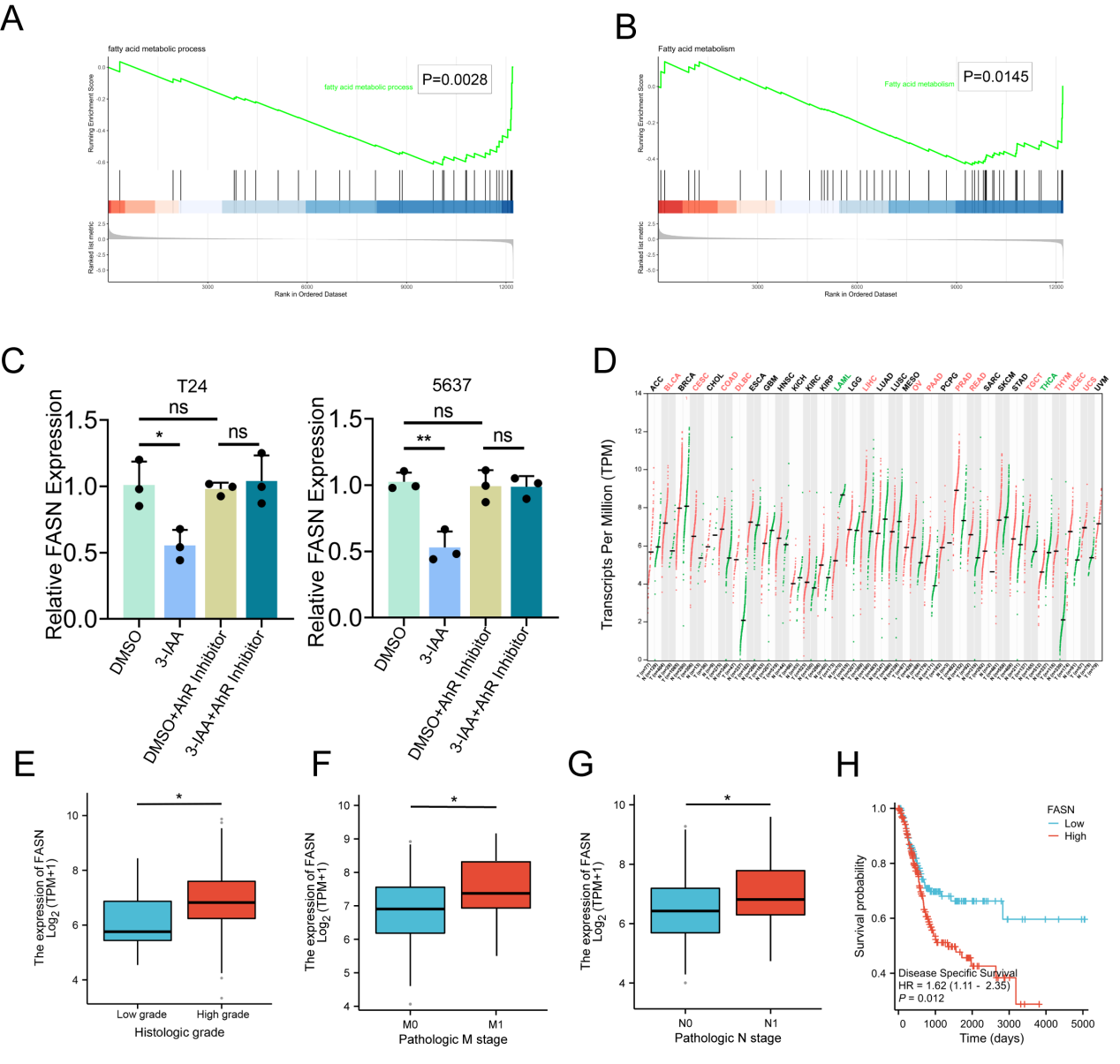
Figure S6**

High FASN expression is associated with poor prognosis in bladder cancer. (A) GSEA results of fatty acid metabolism and (B) fatty acid metabolic process. (C) The relative mRNA expression of FASN in T24 and 5637 cells under 3-IAA treatment, AhR inhibitor treatment, and their combination. (D) Pancancer analysis of FASN expression. (E) Kaplan‒Meier curves for overall survival of BCa patients with high vs. low expression of FASN. (F) Analysis of FASN expression in the low-grade and high-grade groups in the TCGA database. (G) Analysis of FASN expression in the M0 and M1 groups in the TCGA database. (H) Analysis of FASN expression in the N0 and N1 groups in the TCGA database. Data are represented as mean ± SEM. **P* < 0.05, ***P* < 0.01, ****P* < 0.001, ns: not significant. FASN: Fatty Acid Synthase; BCa: bladder cancer; WT: wild-type.


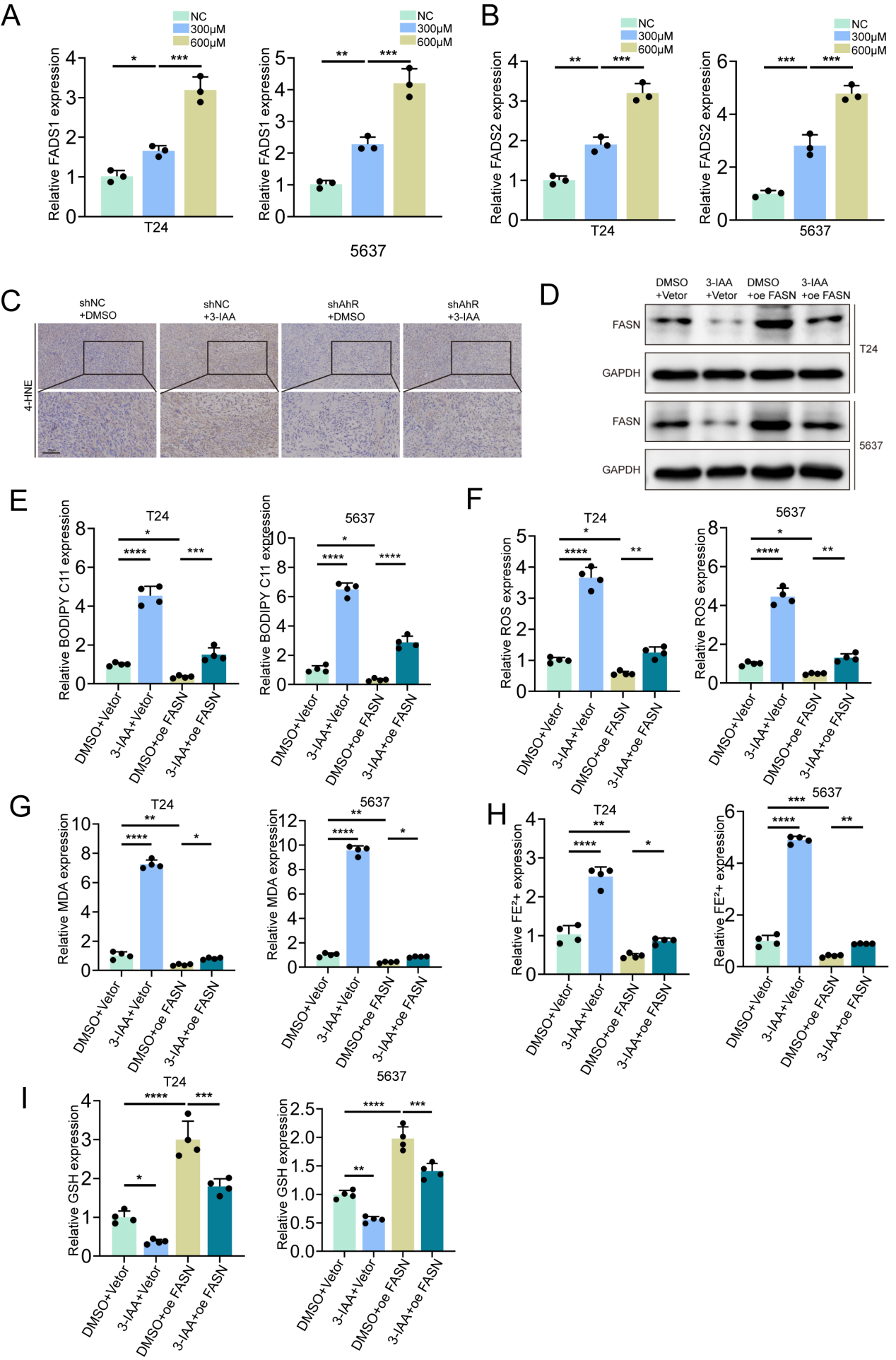


**Figure S7** Overexpression of FASN reversed the increased ferroptosis sensitivity induced by 3-IAA. (A-B) The relative mRNA expression of FADS1 and FADS2 in T24 and 5637 cells treated with different concentrations of 3-IAA (300 μM and 600 μM) or DMSO. (C) Immunohistochemical staining show 4-HNE expression in tumors. (D) Immunoblot analysis of FASN expression in the indicated cells. (E-F) Relative BODIPY C11 and ROS levels were examined in the indicated cells. (G) MDA, (H) Fe2+, and (I) GSH levels were examined in the indicated cells. Data are represented as mean ± SEM. **P* < 0.05, ***P* < 0.01, ****P* < 0.001, ns: not significant.
